# PCDH9 suppresses melanoma proliferation and cell migration

**DOI:** 10.3389/fonc.2022.903554

**Published:** 2022-11-14

**Authors:** Jiaojiao Zhang, Hui-Zhi Yang, Shuang Liu, Md Obaidul Islam, Yue Zhu, Zuhua Wang, RongYi Chen

**Affiliations:** ^1^ Dermatology Department, Dermatology Hospital, Southern Medical University, Guangzhou, Guangdong, China; ^2^ College of Food and Health, Zhejiang Agriculture and Forestry University, Hangzhou, Zhejiang, China; ^3^ The Seventh Affiliated Hospital of Southern Medical University, Foshan, Guangdong, China; ^4^ Department of Surgery, University of Miami, Miami, FL, United States; ^5^ College of Pharmaceutical Sciences, Guizhou University of Traditional Chinese Medicine, Guiyang, Guizhou, China; ^6^ Nano-drug Technology Research Center at Guizhou University of Traditional Chinese Medicine, Guiyang, Guizhou, China

**Keywords:** PCDH9, RAC1, melanoma cell suppression, MMP2, MMP9, CCND1 (Cyclin D1)

## Abstract

**Background:**

Melanoma has dramatically increased during last 30 years with low 5-year survival and prognosis rate.

**Methods:**

Melanoma cells (A375 and G361) were chosen as the *in vitro* model. The immunohistochemical (IHC) analysis and bioinformatics mining exhibited the suppression of PCDH9 on melanoma. The interference and overexpression of PCDH9 were infected by lentivirus. The effects of PCDH9 on melanoma cells were assessed in terms of alteration of PCDH9 such as cell viability, apoptosis, cell cycle, and wound-healing assay. Moreover, expressions of PCDH9 with other genes (MMP2, MMP9, CCND1, and RAC1) were also assessed by PCR.

**Results:**

The alteration of PCDH9 has a negative correlation with MMP2, MMP9, and RAC1 but had a positive correlation with CCND1 (Cyclin D1) and apoptosis. Increase of PCDH9 could suppress melanoma cells and inhibit migration but not exert significant effects on cell cycle. IHC showed lower PCDH9 expression in melanoma tissue with main expression in cytoplasm.

**Conclusion:**

Overexpressed PCDH9 suppressed melanoma cells, and PCDH9 can be considered as an independent prognostic factor for melanoma; even re-expression of PCDH9 can serve as a potential therapeutic strategy for melanoma treatment.

## Graphical Abstract

Proposed model for the role of PCHD9 regulates melanoma. Increase of PCDH9 suppressed NADPH oxidase activity, decreased ROS generation, and ROS-induced angiogenesis. PCDH9 can target complex-bound Rac1 to weaken angiogenesis by regulating NADPH oxidase, ROS production, and DNA damage susceptibility through cyclin D1 trafficking. VEGF binding to VEGFR2 leads to activating and translocating RAC1 into the plasma membrane. Whereas ROS-dependent signaling events may trigger angiogenesis (i.e., cell migration and proliferation) and influence MMP2 that affect growth factor and tumor promoter stimulation as well.

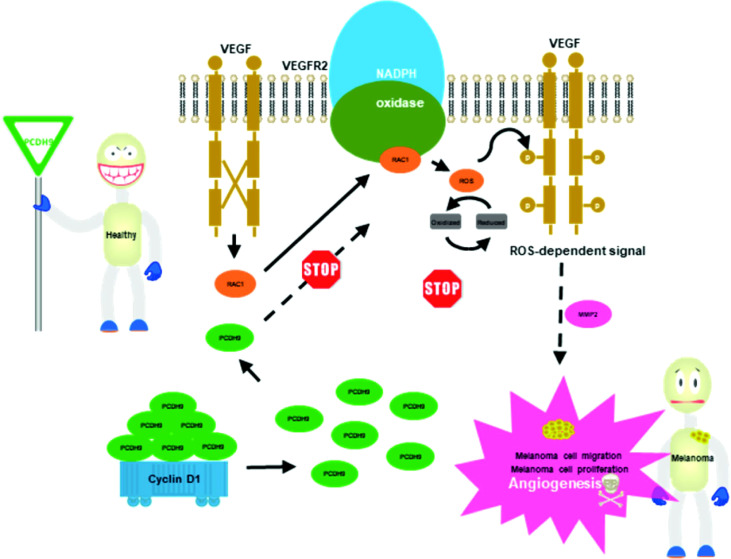

## Introduction

Cutaneous melanoma, a type of skin cancer, develops in melanocytes, which takes up to 2% of all cancer deaths globally (1). The incidence rates of cutaneous melanoma are quite different among countries: lower incidence in Asia than in the West due to genetic sensitivity responses among populations. According to WHO’s Global Health Estimates, there are 0.43–0.48 new cases per 100,000 people in East and Southeast Asia, whereas 12.6–18.8 new cases per 100,000 people in North America and Europe each year (1). The prognosis of melanoma varies in different diagnostic stages: a 5-year survival rate of 98% for patients with non-metastatic cutaneous melanomas compared with 62% and 16% for patients suffering regional and distant metastatic melanoma, respectively (2). The melanoma characters of Asian and European population are different in subtypes frequencies, risk factors, and mutation patterns (3). Although the incidence rate of cutaneous melanoma is lower in the Asian population, the mortality rate is higher and commonly with poorer prognosis (4). Therefore, the existing studies based on the Caucasian populations are not suitable for melanoma in Asian countries. For the above reason, we conduct the study of melanoma inhibition by resveratrol and found that this natural product can suppress A375 (a melanoma cell line) along with protein expression fluctuation (PCDH9, RAC1, and Cyclin D1) (5). On the basis of this, the Chinese patients’ skin biopsy was assessed by immunohistochemistry (IHC) following the work by Dinehart et al. (6), in which the varieties of protocadherin 9 (PCDH9) expressions were found among patients’ skin, normal skin, and pigmented nevus tissue.

PCDH9 belongs to protocadherin, which constitutes the largest subfamily of cadherin group (including type I classical cadherins, type II atypical cadherins, desmosomal cadherins, flamingo cadherins, and protocadherin) (7). The protocadherin subfamily is calcium-dependent cell-cell adhesion molecules that revealed six extracellular cadherin repeats with conserved calcium ion–binding domain (8). The focused PCDH9, a member of δ1-subfamily (including PCDH1, PCDH7, PCDH9, and PCDH11), is involved in cell adhesion establishment and disruption (9). Previous studies have revealed a strong correlation between δ-PCDHs and tumor suppressor, along with the low expressions of δ-PCDH that correlate with poor prognosis. Meanwhile, studies found that δ-PCDH inhibits tumor cell proliferation by regulating cell proliferation (10). In addition, studies found that the overexpression of PCDH9 could suppress different cancers (11, 12) and tumor cells by arresting cell cycle at G0/G1 phase (13, 14). However, scarce data of PCDH9 focus on inhibiting melanoma. Moreover, the role of P-cadherin behaves differently depending on tumor cell context (15). Interestingly, melanoma cells represent unique response to cadherins. Unlike tissues like bladder (16, 17), the effective role of P-cadherin exhibits suppressive behavior on melanoma, whose membranous expression decreased at the metastatic stage (18, 19). RAC1, a GTPase, has been studied profoundly as a conserved member of RHO family and has been recognized as a central signaling hub for oncogene transforming. Meanwhile, some investigations discover its activating mutations in malignancies especially malignant melanoma (20). In addition, RAC1 expression correlates with melanocyte proliferation and can evade immune checkpoint (21). RAC1 also plays important roles in tumor biology by modulating cell processes (22, 23). Hence, RAC1 is a good indicator to reflect the effect of PCDH9 on melanoma. RAC1 functions as a molecule switch between active guanosine triphosphate (GTP)-bound and inactive guanosine diphosphate (GDP)-bound states through conformation changes closed to the nucleotide-binding site (7). RAC1 could affect cellular adhesion, migration, and invasion (24), and it plays important roles in tumor biology by modulating cell processes (22, 23). Furthermore, the activities of RAC1 have been reported to involve different stages of oncogenesis, such as initiation, progression, invasion, and metastasis (25), even it was ranked as the third most frequently occurring mutation in melanoma induced by UV (26, 27). In addition, some reported reactive oxygen species (ROS) involve in tumor cell migration and invasion (28, 29), and a key component of NAPDH-oxidase complex is formed by RAC1, one of the major enzymatic sources of ROS in various tissues (30). However, it is reported that RAC1-dependent nicotinamide adenine dinucleotide phosphate (NADPH) oxidase complex is involved in endothelial migration by mediation of angiotensin-1 (Ang-1) and vascular endothelial growth factor (VEGF) (31, 32). As known, the endothelial migration is essential for tumor cell invasion, where RAC1–NADPH oxidase complex induce expression of matrix metalloproteinases (MMPs) after growth factor and tumor promoter stimulation (33, 34). MMPs are involved in extracellular matrix (ECM) regulation, which is important in the maintenance of microenvironment and homeostasis (35). MMP2 and MMP9 belonging to MMPs are classified as gelatinases. Moreover, several studies demonstrated the important role of MMPs in melanoma (35). MMP2 has high expression levels in primary nodular melanoma that is the predominant subtype in Yogyakarta, Indonesia (1, 36). In addition, MMP2 has been believed to act as a pro-tumorigenic and pro-metastatic factor in different cancers including melanoma (37), whereas MMP9 shares the similar effect on tumor that can reconstruct the ECM to make tumor invasive process easier along with highest presence in tumor development (including melanoma). MMP9 has also been considered as an indicator of invasiveness in malignant melanoma and a marker of treatment by BRAF (B-Raf proto-oncogene, serine/threonine kinase) inhibitors, a common genetic mutation in melanoma (38). Hence, MMP2 and MMP9 were chosen as tumorigenic indicators to exhibit the correlation between PCDH9 and melanoma suppression. CCND1 encodes Cyclin D1 protein that belongs to highly conserved cyclin family that exhibits periodicity abundance throughout cell cycle. Cyclin D1–CDK4 complex regulates cell cycle during G1/S transition. Cyclin D1 is the component of ternary complex (Cyclin D1/CDK4/CDKN18) and is required for the Cyclin D1–CDK4 complex translocation. CCND1 was selected to compare with the cell cycle assay due to our previous investigation of melanoma inhibition by resveratrol (5).

According to previous studies of ours and others, the main objective of this investigation is to clarify the role of PCDH9 in melanoma and to provide evidence and a novel possible treatment of melanoma. Certain assays (cell viability, apoptosis and cell cycle assays, and PCR) were performed to explore the alteration influence of PCDH9 in melanoma cells. Currently, we found that the following: 1) overexpression of PCDH9 could suppress melanoma cells and inhibit migration; 2) the alteration of PCDH9 had a negative correlation with MMP2, MMP9, and RAC1 but positive correlation with CCND1 (Cyclin D1) and apoptosis; 3) although the cell regulator gene, CCND1 (Cyclin D1) altered with PCDH9 but did not exert significant effects on cell cycle; and 4) the IHC results exhibited the lower positive percentage of PCDH9 expression in human melanoma tissue than in normal skin or/and pigmented nevus tissue, and IHC also showed the PCDH9 expression in melanoma tissue and mainly expressed in the cytoplasm. It suggests that Cyclin D1 (CCND1) could affect tumorigenesis by mechanism of nuclear trafficking (39) but not *via* cell regulating. Together, our results reveal that the alteration of PCDH9 expression could suppress melanoma proliferation and cell migration.

## Material and methods

### Chemical and antibodies

Dulbecco’s modified Eagle’s medium (DMEM), fetal bovine serum (FBS), phosphate buffer solution (PBS) (pH = 7.2), Diethyl Pyrocarbonate (DEPC)-treated water (Ambion), and TRIzol reagent (Invitrogen) were purchased from Gibco (Thermo Fisher Scientific, Shanghai, China); ethanol (70%), isopropyl alcohol, and Triton X-100 were bought from Sigma-Aldrich (Shanghai, China); Cell Counting Kit-8 (CCK-8) was bought from Dongren Chemical Technology (Shanghai, China); GV358-PCDH9 lentivirus and GV358-siRNA (short interfering RNA) lentivirus were designed by GeneChem (Shanghai, China); SYBR^®^ Premix Ex Taq™ Ex Taq ™ II and PrimeScript™ RT reagent Kit with gDNA Eraser were bought from Takara Bio Inc. (Beijing, China); water was obtained from EPED-20TF (Nanjing, China).

### Cell culture

Both cell lines A375 and G361 (ATCC^®^ CRL-1619™) were bought from the American Type Culture Collection (ATCC) (MD, USA). They were grown in DMEM supplemented with 10% heat-inactivated FBS as well as penicillin (100 IU/ml) and streptomycin (100 μg/ml). Cells were maintained in a CO_2_ incubator at 37°C under a humidified atmosphere (95% air, 5% CO_2_).

### Sample collection and preparation

Tissues [human normal skin tissue (n = 45), human pigmented nevus (n = 30), and primary malignant melanoma tissue (n = 30)] were collected and prepared as paraffin specimens until use. These tissues were ethically acquired from the outpatient clinic of the Affiliated Hospital of Guangdong Medical University with Chinese population (Han people) with personal identifiers redacted. The protocol of biopsy was proceeded according to the Ethical Committee of Guangzhou Medical University (PJ2015055KT).

### Immunohistochemical stains

The paraffin specimens were deparaffinizated including two 100% xylene changes (xylene I, 10 min; xylen II, 10 min) followed by rehydration with a graded series of ethanol (anhydrous ethanol I, 5 min; anhydrous ethanol II, 5 min; 95%, 85%, and 75% ethanol, 5 min each) and then rinsed under distilled running water for 3–5 min. Antigen retrieval consisted of a 2-min incubation of slides in citric acid retrieval solution heated to 98°C with a commercial steamer following a cool down step to room temperature (cold water and ice pack were added), slides were transferred into a wet box and were then rinsed three times with PBS. After protein blocking, primary antibodies (1:200) (anti-PCDH9, Sigma-Aldrich; lot #; HPA015581) were incubated at 4°C overnight. After being in room temperature for 30 min, the slides were washed three times with PBS for 3 min each. After removing PBS and protein blocking, secondary antibodies (1:1,000) were added at room temperature for 1 h. The slides were then washed three times with PBS for 3 min each. After removing PBS, one drop of the prepared Diaminobenzidine (DAB) solution (1 ml A:1 drop B:1 drop C) for DAB staining was added, and the slides were observed under a microscope. After being rinsed in running water for 10 min, hematoxylin was added for 1 min, and then, the slides were washed by water for 5 min. The slides were then dehydrated in a series of ethanol (75%, 85%, 95%, and 100%) and 100% xylene changes and mounted with a coverslip with dry neutral resin.

### Evaluation of various protein expressions in MM

Various protein expressions in MM were evaluated by semi-quantitative analysis, according to the staining intensity and the percentage of positive cells. The score standards of staining intensity were as follows: no coloration, 0; low intensity (light yellow), 1; medium intensity (light brown), 2; and high intensity (dark brown), 3. Five fields of view were randomly selected under a microscope (×400), and 500 cells were counted as one unit; meanwhile, the percentage of positive cells was calculated. The percentage scores were as follows:<5%, 0; 6%–25%, 1; 26%~50%, 2; 51%~75%, 3; and >75%, 4. The score standards were the product of staining intensity and percentage of positive cells: 0, negative (−); 1 to 4, positive (+); 5 to 8, moderately positive (++); and 9 to 12, strongly positive (+++).

### Survival analysis

Gene Expression Profiling Interactive Analysis (GEPIA) is web server for comprehensive expression analyses (40). This web-based tool is based on The Cancer Genome Atlas (TCGA) (41) and Genotype-Tissue Expression (GTEx) (42). The GEPIA web server provides survival analysis. GEPIA was used to analyze the tumor metastasis indicators of this study, i.e., MMP2.

### Transfection

Melanoma cells (A375 and G361) were seeded in six-well plates (1 × 10^5^ cell per well) the day before transfection and were transfected by two types of lentiviruses (siRNA and PCDH9) (S3). Control groups were transfected with the empty vector. Blank groups were treated with transfection reagent only. Transfection was performed using GeneChem Transfection Reagent (Shanghai, China), according to the manufacturer’s instructions. Seventy-two hours after transfection, cells were observed by a fluorescent inverted microscope as screened by puromycin. The efficiency of PCDH9 alteration in melanoma cells was detected by real-time PCR.

### Cell viability by Cell Counting Kit-8

Cells were seeded into 96-well plates at a density of 2 × 10^5^ cells per well and treated by non-transfected plasmid, transfected with empty plasmid, and transfected with PCDH9-overexpressed plasmid as explained above. After incubation at 24, 48, 72, and 96 h, 10 μl of CCK-8 was added to each well, and cells were incubated for another 4 h at 37°C. The level of colored formazan derivative was analyzed on Thermo Scientific Multiskan FC (Vantaa, Finland) at a wavelength of 450 nm. The viable cells were directly proportional to the formazan production, and the percentage of viable ones was calculated. Equations 1 and 2 were utilized to determine the viability rate and inhibition rate, respectively


(Equation 1) $$V\%=\frac{As-Ab}{Ac-Ab}\times 100\%$$

(Equation 2) $$I\%=\frac{Ac-As}{Ac-Ab}\times 100\%$$

V%: the viability rate;

A_s_: the absorbing values of experimental wells (cells with medium, CCK-8, and PCDH9-overexpressed plasmid);

A_b_: the absorbing values of blank wells (medium, CCK-8, and empty plasmid);

A_c_: the absorbing values of control wells (cells with medium and CCK-8);

I%: the inhibition rate.

### Apoptosis detection by flow cytometer

Apoptosis was analyzed by cytometric analysis, using FITC Annexin V Apoptosis Detection Kit (BD, USA). Cells were seeded in six-well plate at a density of 1 × 10^6^ cells per well. Briefly, cells were treated with Camptothecin stock solution (10 mg of lyophilized powders were dissolved in 2.87 ml of Dimethyl Sulfoxide (DMSO) to make 10 mM stock solution; 1 μl was used) and incubated for 5 h at 37°C. After that, the cells were centrifuged (1,000 rpm for 10 min), washed twice with cold PBS, and resuspended in 1× binding; 5 μl of FITC Annexin V and 5 μl of PI (Bio-Rad) were added to cell suspension, incubated, and protected from light for 15 min at room temperature. Finally, samples were analyzed using the BD FACS Canto II flow cytometer.

### Cell cycle assay

Cell cycle was analyzed using the flow cytometry. Briefly, cells were seeded in six-well plates at a density of 1 × 10^6^ cells per well. Cells were then detached, centrifuged at (1,000 rpm for 10 min), and then vortexed with 5 ml of cold 75% ethanol. Cells were incubated at −20°C for 2 h and washed twice with PBS to remove ethanol. Cells were resuspended in 0.5 ml of PI/RNase staining buffer (Bio-Rad) for 15 min at room temperature; samples were analyzed using the BD FACS Canto II flow cytometer.

### Wound-healing assay

Cells were seeded into six-well plates at a density of 1.5 × 10^5^ cells per well until confluency of 80%–100% is reached and then scratched by a sterile 10-μl pipette tip. Cells were washed twice with PBS; then, a complete medium was added to allow cells moving into the gap and photographed by using an inverted microscope DMI3000B (Leica, Germany) at 0, 24, and 48 h. ImageJ (MD, USA) was used to measure the wound space. Migration rate was calculated as the proportion of initial scratch distant of each sample and the mean distance between the borderlines of the remaining free cells after migration.

### Quantitative real-time PCR analysis

Each frozen pellet of melanoma cells (A375 and G361), treated in different experimental conditions, was homogenized in a lysis buffer. Total RNA was isolated through the TRIzol Reagent Total RNA isolation system (Thermo Fisher Scientific, USA) according to the manufacturer’s reference guide. Total RNA was quantified by nanodrop and was reverse-transcribed by a PrimeScript RT reagent kit (TaKaRa) and referred to SYBR Green qPCR assay introduction (SYBR^®^ Premix Ex TaqTM II kit) by MasterCycler Gradient PCR (Thermo Fisher Scientific, USA). The reaction mixture (20 μl) was taken and incubated for 3 min at 95°C. Quantification of genes was performed with the 2^−ΔΔCT^ method, as described previously (43): The sample was cycled (95°C, 10 s; 60°C, 20 s) for 40 times by the ABI7500Fast Real-time PCR System Amplifier (Thermo Fisher Scientific, USA). The primers designed for selected genes (PCDH9, CCND1, MMP2, MMP9, and RAC1) and amplicon sizes are shown in **Supplementary Table S1**.

### Statistical analysis

Data are shown as means ± SEM from at least three independent experiments. Two-tailed Student’s t-test was used to compare differences between two groups. One-way ANOVA followed by least significant difference *post hoc* tests was used to compare differences among three or more groups (Originlab 2020, Northampton, MA, USA). A value of *p*< 0.05 was considered statistically significant, whereas a value of *p<* 0.01 was considered highly statistically significant. *p< 0.05, **p< 0.01, and ***p< 0.001.

## Results

### PCDH9 protein expressed differences in normal skin, pigmented nevus, and melanoma tissue tested by IHC stains

**Figure 1:**
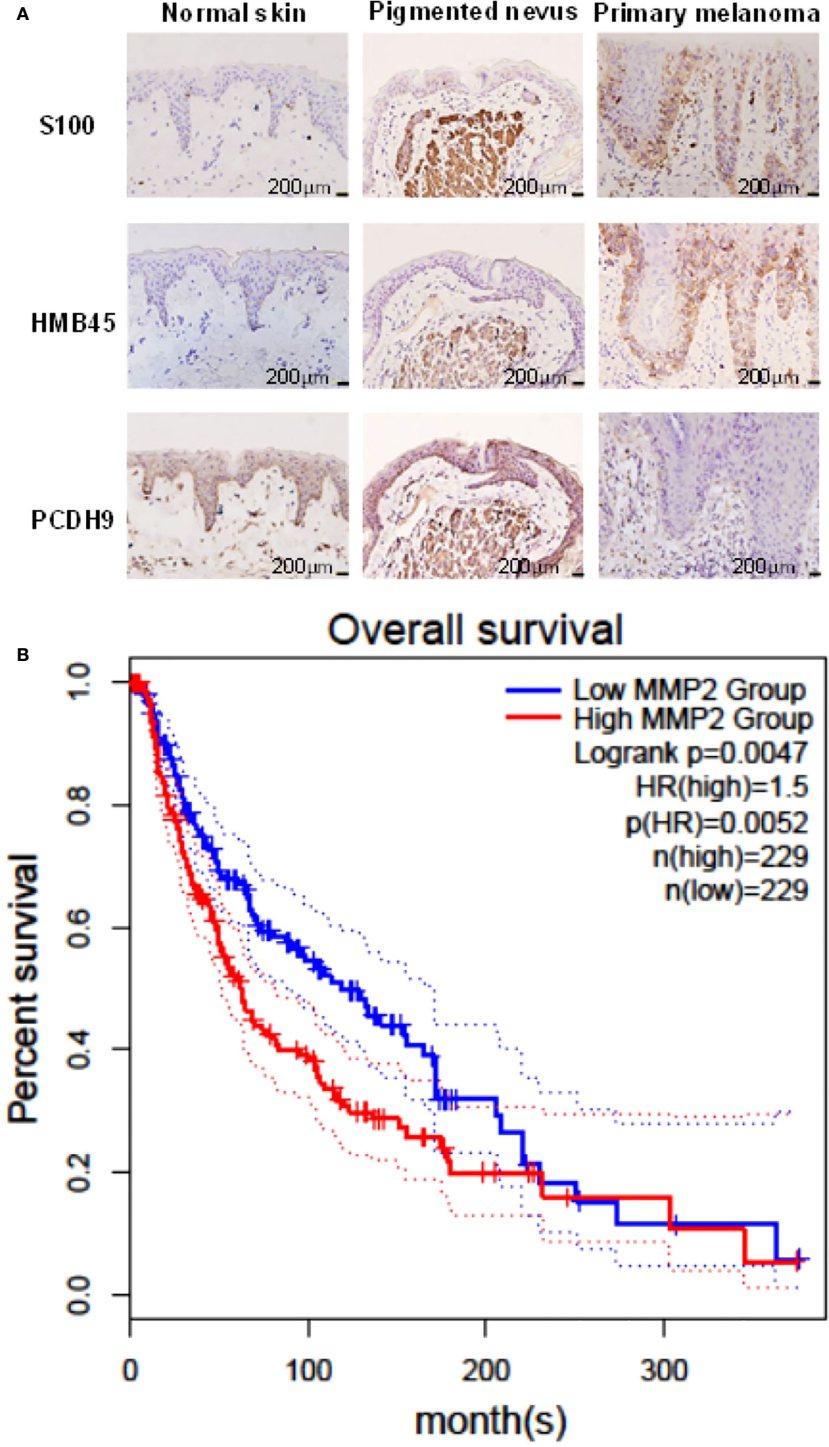
**(A)** Immunohistochemical analyses of PCDH9 expression in normal skin, pigmented nevus, and melanoma tissue. Positive percentage of PCDH9 expression was lower in human melanoma tissue than in normal skin or/and pigmented nevus tissue. PCDH9 was mainly expressed in cytoplasm but a small amount in nuclei. S100 and HMB45 are melanoma markers. The scale bar represents 200 μm. **(B)** Survival curves of MMP2 in normal and skin cutaneous melanoma (SKCM) tissues based on TCGA data in GEPIA. Red line represents the samples with MMP2 highly expressed (n = 229), whereas blue line exhibits lowly expression (n = 229) (log rank, *p* = 0.0047). HR represents hazard ratio. The *p*-value of HR is less 0.05 (*p* = 0.0052).

IHC results showed that the positive percentage of PCDH9 expression was lower in human melanoma tissue than in normal skin or/and pigmented nevus tissue; in addition, PCDH9 was mainly expressed in the cytoplasm, whereas a small amount was expressed in the nuclei. A positive percentage of PCDH9 was expressed in normal skin or/and pigmented nevus tissue but only 23.3% (7 of 30) in melanoma tissue, which was lower than that in non-tumor tissue **(** **Table 1**, **Figure 1A** **)**. The IHC results are consistent with the studies of δ-PCDHs that include PCDH9 and are involved in cell adhesion establishment and disruption. Moreover, δ-PCDHs are demonstrated as tumor suppressors by regulating cell proliferation, and the lower expressions of δ-PCDH have poorer prognosis (10). Moreover, the expression of PCDH9 was significantly lower in high-grade and worse histological type of tumors of glioma, gastric, and prostatic cancers (44, 45).

**Table 1:** The positive percentage of PCDH9 expression in normal skin, pigmented nevus, and melanoma tissues.

Type	Total	PCDH9 (−)	PCDH9 (+)	Positive Percentage
Normal skin	45	0	45	100.0%
Pigmented nevus	30	0	30	100.0%
Melanoma	30	23	7	23.3%

### The survival analysis of MMP2

GEPIA was used for survival analysis of MMP2: The cutoff was set as median; the hazards ratio are calculated based on Cox pH model; all datasets were selected (BRAF Hotspot Mutants, NF1 Any Mutants, RAS Hotspot Mutants, Triple WT). The survival rate of highly expressed MMP2 is poorer than that of lowly expressed MMP2, and the HR is 1.5 (*p<* 0.05) **(** **Figure 1B** **)**. The result of MMP2 survival analysis revealed the positive correlation between MMP2 and poor prognosis of melanoma, and it is consistent to the previous studies (1, 35) due to pro-tumorigenic and pro-metastatic effects of MMP2 (37).

### PCDH9 expression affected selected genes expression

**Figure 2:**
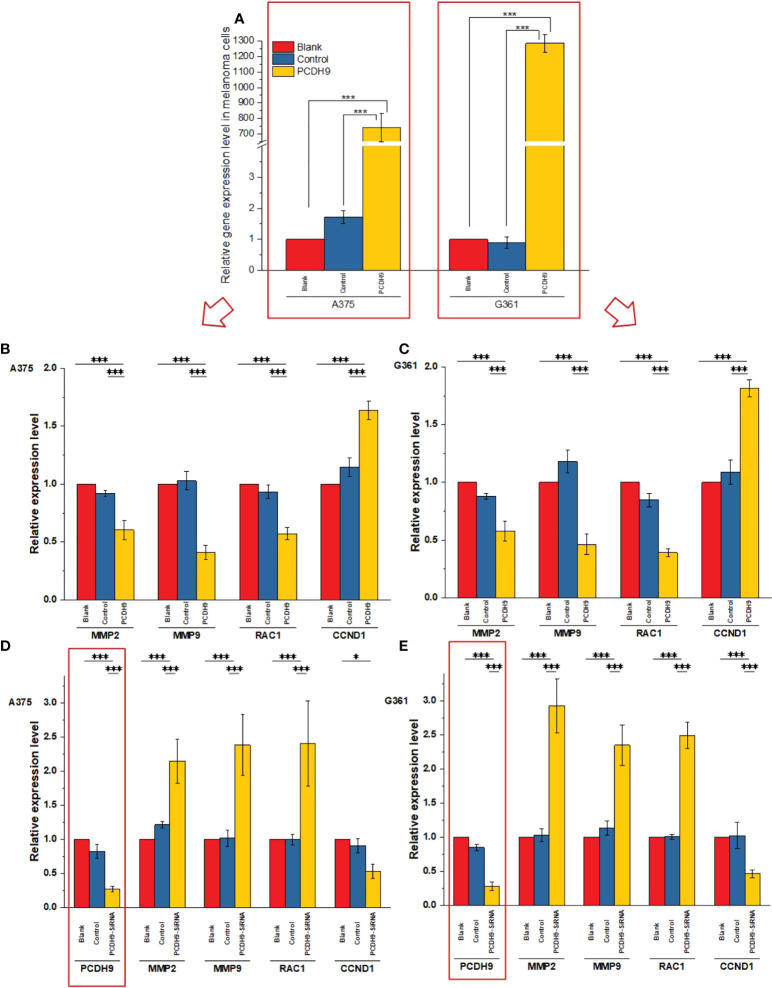
Effects of overexpressed and interfered PCDH9 in melanoma cells measured by PCR analysis **(A)**. The expressions of PCDH9 were significantly upregulated by lentivirus infection. Overexpressed PCDH9 significantly upregulated CCND1 and downregulated RAC1 and MMP9 in both cells [A375 **(B)** and G361 **(C)**]. Interfered PCDH9 downregulated CCND1 and upregulated RAC1 and MMP2 in both cells [A375 **(D)** and G361 **(E)**]. *p < 0.05, and ***p < 0.001 compared in groups by using one-way ANOVA followed by least significant difference *post hoc* tests.

PCDH9 was overexpressed by lentivirus with PCDH9 plasmid **(** **Figure 2A** **)** and interfered by lentivirus with siRNA **(** **Figures 2D, E** **)**. The relative expression of selected genes (CCND1, MMP2, and RAC1) varied with PCDH9 expression, but the effectiveness on them was different. PCDH9 and CCND1 (Cyclin D1) exhibited a positive correlation **(** **Figures 2B–E** **)**, whereas MMP2, MMP9, and RAC1 exhibited a negative correlation with both melanoma A375 and G361 cells **(** **Figures 2B–E** **)**.

### Effects of overexpressed PCDH9 on cell viability

**Figure 3:**
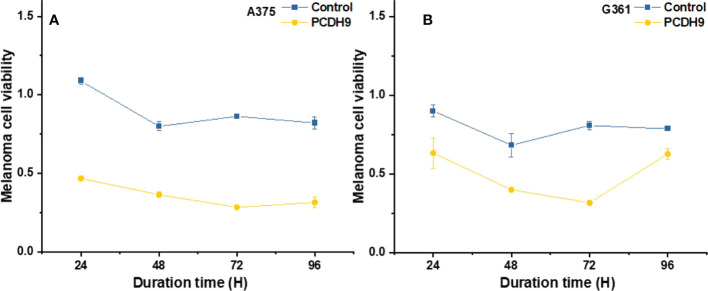
The viability of melanoma cells significantly was reduced by overexpressed PCDH9 in A375 **(A)** and G361 **(B)** cells. The alteration of PCDH9 expression significantly affected the apoptosis of melanoma cells by using one-way ANOVA followed by least significant difference *post hoc* tests.

The overexpression of PCDH9 reduced the proliferation of melanoma cells. Overexpressed PCDH9 groups showed indeed a lower viability than control groups **(** **Figure 3** **)**. As time passed, the viability of melanoma cells tended to stabilize, but PCDH9-overexpressed groups had less viable cells than control groups in different durations, and the differences between PCDH9 and control groups were significant (24, 48, 72, and 96 h) **(** **Figures 3A, B** **)**.

### Effects of PCDH9 alteration on apoptosis and cell cycle

**Figure 4:**
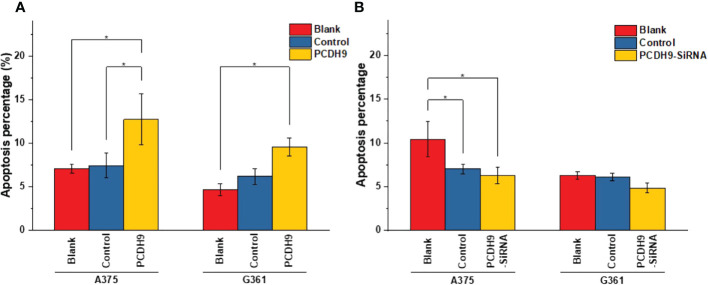
The apoptosis percentage of PCDH9 overexpression was exhibited by **(A)** A375 and **(B)** G361. The overexpression of PCDH9 significantly promoted apoptosis in both cell lines. *p< 0.05 compared in groups by using one-way ANOVA followed by least significant difference *post hoc* tests. The interference of PCDH9 reduced apoptosis in A375 cell line and in a more modest manner in G361 cell lines. *p < 0.05 compared in groups by using one-way ANOVA followed by least significant difference *post hoc* tests.

**Figure 5:**
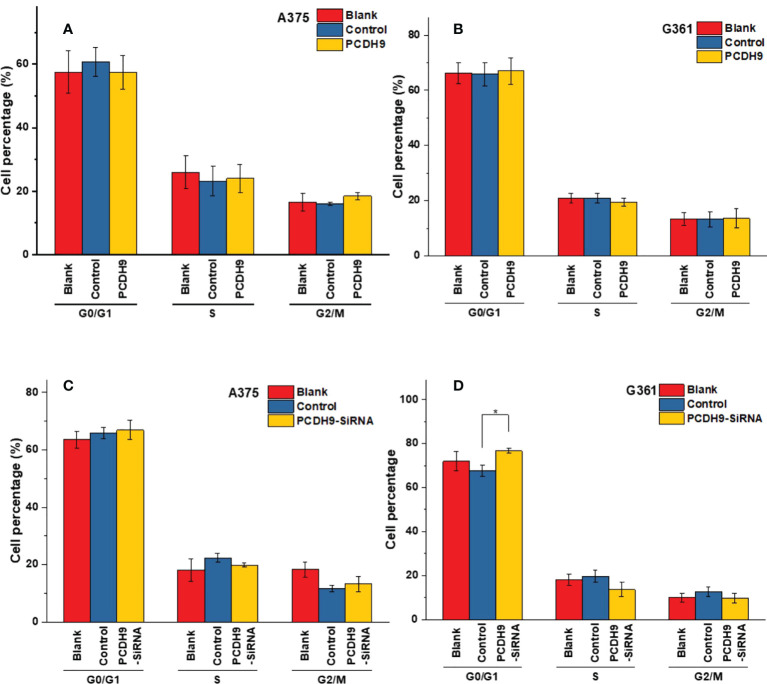
The varieties of PCDH9 expression did not significantly affect melanoma cell regulation. The cell percentage of melanoma cells affected by overexpressed PCDH9 in different cell period time in A375 **(A)** and G361 **(B)** cell lines. The cell percentage of melanoma cells affected by PCDH9 interference in different cell period time in A375 **(C)** and G361 **(D)** cell lines. *p < 0.05 compared in groups by using one-way ANOVA followed by least significant difference *post hoc* tests.

The apoptosis percentage of PCDH9 overexpression was exhibited by **Supplementary Figures S1A** (A375) and **Supplementary Figures S1B** (G361), whereas the apoptosis percentage of PCDH9 interference was exhibited by **Supplementary Figures S1C** (A375) and **Supplementary Figures S1D** (G361). The cell cycle percentage of PCDH9 overexpression was exhibited by **Supplementary Figures S2A** (A375) and **Supplementary Figures S1B** (G361), whereas the cell cycle percentage of PCDH9 interference was exhibited by **Supplementary Figures S2C** (A375) and **Supplementary Figures S1D** (G361). The overexpression of PCDH9 promoted the apoptosis in both melanoma cells **(** **Figure 4A** **)**, whereas the interfered PCDH9 barely influenced the apoptosis in both cell lines **(** **Figure 4B** **)**. The alteration of PCDH9 and apoptosis exhibited a positive correlation **(** **Figure 4** **)**. Regarding the cell cycle arrest, there was no discrepancy between overexpressed PCDH9 or interfered PCDH9 groups and other groups (blank and control groups) in both cell lines (A375 and G361) **(** **Figures 5A–D** **)**. Cyclin D1, encoded by CCND1, is the component of ternary complex (Cyclin D1/CDK4/CDKN18) that can regulate cell cycle during G1/S transition, but the changes of PCDH9 did not affect cell cycle. The results revealed that PCDH9 may affect melanoma cells by different ways.

### Effects of overexpressed PCDH9 on wound healing

**Figure 6:**
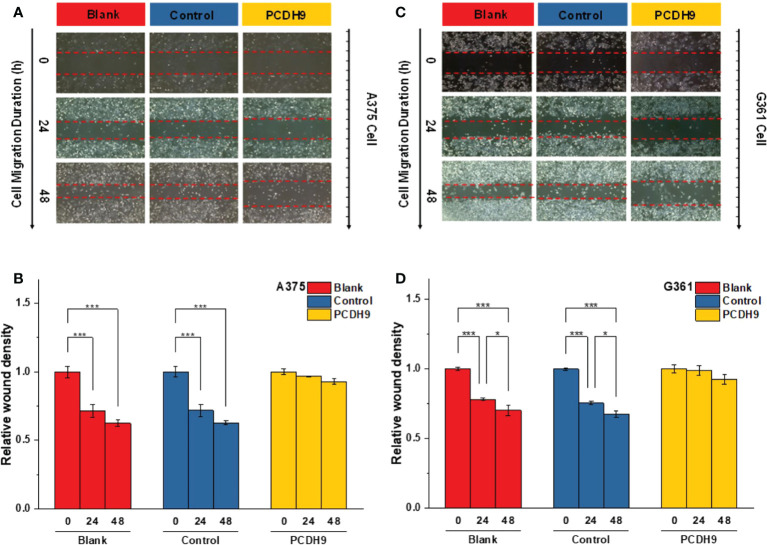
The scratched boundary of migrator cells was observed by inverted microscope DMI3000B (Leica, Germany). Representative image of melanoma cells invaded from the scratched boundary in A375 **(A)** and G361 **(C)** cell lines. Relative density of scratched boundary was not altered in overexpressed PCDH9 groups comparing with blank and control groups in A375 **(B)** and G361 **(D)** cell lines. *p < 0.05, and ***p < 0.001 compared in groups by using one-way ANOVA followed by least significant difference *post hoc* tests.

With respect to cell migration, after quantifying the scratched boundary by ImageJ **(** **Figures 6A, C** **)**, the results revealed that the relative density decreased with the duration of cell culture in the blank and control groups (*p<* 0.001), whereas the relative wound density did not significantly change in the overexpressed PCDH9 groups (*p >* 0.05) **(** **Figures 6B, D** **)**. The relative wound density of scratched boundary was significantly different in the overexpressed PCDH9 groups compared with the blank and control groups after 24 and 48 h (*p<* 0.001) **(** **Figures 6B, D** **)**. The wound did not heal so much, when PCHD9 were overexpressed **(** **Figure 6** **)**.

## Discussion

Specimen investigations of IHC assay exhibited lower PCDH9 expressions in malignant melanoma specimens than in benign nevus tissue or/and normal skin. Moreover, our study revealed that PCDH9 was mainly expressed in the cytoplasm rather than in nuclei. This result is consistent with other cancers like glioma, gastric, and prostatic, in which lower expression of PCDH9 was observed in high-grade and worse histological type of tumors (44, 45). The survival analysis of MMP2 associated the high expression with the lower survival rate and the low expression with the higher survival rate. The HR was 1.5 (*p<* 0.05). Our study agreed with the previous investigations that MMP2 and MMP9 can represent a biomarker of malignant melanoma (37), and downregulating MMP2 expression would increase prognostic survival. We explored and performed a series of investigations, including cell viability assay, apoptosis assay, and PCR of PCDH9 alteration by lentivirus (GV358-PCDH9 and GV358-SiRNA) in A375 and G361. According to our results, overexpressed PCDH9 upregulated expressions of CCND1, whereas MMP2, MMP9, and RAC1 were downregulated. Interfered PCDH9 induced downregulation of CCND1, whereas MMP2, MMP9, and RAC1 were upregulated. The results agreed with the previous studies that the lower expression of PCDH9 was associated with the worse mean survival rate (44, 45). The alteration of PCDH9 exhibited positive correlation with apoptosis that the apoptosis was promoted with overexpressed PCDH9 but decreased with interfered PCDH9. The overexpression of PCDH9 reduced the viability of melanoma cells. Our results agreed with recent studies that found lower PCDH9 expression in various cancer types (41, 46, 47). The alteration of PCDH9 affected CCND1 (Cyclin D1), the cell regulator protein. The previous investigations of hepatocellular carcinoma (HCC) found that PCDH9 suppresses HCC cells by inducing cell cycle arrest at G0/G1 phase (13). Our results suggested that the effect of PCDH9 on melanoma by RAC1 suppresses RAC1-dependent NADPH oxidase activity to decrease ROS generation and ROS-induced angiogenesis. PCDH9 can target complex-bound RAC1 to weaken angiogenesis by regulating NADPH oxidase, ROS production, and DNA damage susceptibility through cyclin D1 trafficking. VEGF binding to VEGFR2 leads to activating and translocating RAC1 into the plasma membrane, whereas ROS-dependent signaling events may trigger angiogenesis (i.e., cell migration and proliferation) and influence MMP2, which affect growth factor, tumor promoter stimulation, and prognostic survival as well. Moreover, RAC1 could affect cellular adhesion, migration, and invasion as well (24). However, the alteration of PCDH9 expression did not affect melanoma cell regulation in a significant manner (*p >* 0.05). This result suggests that PCDH9 and Cyclin D1 (CCND1) could affect melanoma cell by different mechanisms. Cyclin D1 (CCND1) could affect tumorigenesis *via* nuclear trafficking (44), which resulted in PCDH9 mainly expressed in the cytoplasm but not in the nucleus. The results of wound-healing assay revealed that the overexpression of PCDH9 could inhibit the cell migration or the duration, which similar to PCDH9 affecting on HCC (48). Recently, Gross et al. found the role of store-operated Ca^2+^ entry (SOCE) in melanoma metastasis that the suppression of Ca^2+^ signaling worsened the melanoma progression and that the concentration of extracellular Ca^2+^ could play the important role (49). Unlike most tissues, melanocytes grow within the extracellular Ca^2+^; in contrary, non-native tissues will be tolerated at high concentration of extracellular Ca^2+^ (49). In the context of SOCE role, we speculate that the binding between PCDH9 and calcium ion can increase the adhesion of melanocytes, whereas the adhesion of non-native cells increases in lower expressions of PCDH9 that can enhance the migration of melanoma cells. To conclude, the increase of PCDH9 could suppress melanoma cells by observing the deregulation of MMP2, MMP9, and RAC1. Although the alteration of PCDH9 could influence CCND1 but not the cell cycle, which suggested it may affect melanoma cells by other mechanisms, such as SOCE combined melanoma cell migration, RAC1-dependent NADPH oxidase correlated with GTP-GDP switch. In summary, PCDH9 can be considered as an independent prognostic factor for melanoma, and re-expression of PCDH9 can serve as a potential therapeutic strategy for melanoma treatment.

## Data availability statement

The datasets generated during and/or analyzed during the current study are available from the corresponding author upon reasonable request.

## Ethics statement

The protocol of biopsy was proceeded according to the Ethical Committee of Guangzhou Medical University (PJ2015055KT). The patients/participants provided their written informed consent to participate in this study. Written informed consent was obtained from the individual(s) for the publication of any potentially identifiable images or data included in this article.

## Author contributions

Conceptualization: JZ, RC, and ZW. Data curation: JZ, YZ, and SL. Formal analysis: JZ, H-ZY, SL, and MI. Project administration: RC and ZW. Resources: RC. Visualization: JZ. Writing original draft: JZ. Review and editing: JZ, RC, and MI. All authors contributed to the article and approved the submitted version.

## Funding

This research was supported by the Natural Science Foundation of Guangdong Province of China (2016A030313682 and 2020A1515010281).

## Acknowledgments

The authors thank Maurizio Battino and Francesca Giampieri (Department of Clinical Sciences, Faculty of Medicine, Polytechnic University of Marche, Ancona, Italy) and Gianluca Storci (Department of Experimental, Diagnostic, and Specialty Medicine, University of Bologna, Italy) for their insight and helpful discussion. We also thank Dr. Jingquan He (Shanghai Biotree Biotechnology, Ltd., Shanghai, China) for his company and his support in protein complex immunoprecipitation.

## Conflict of interest

The authors declare that the research was conducted in the absence of any commercial or financial relationships that could be construed as a potential conflict of interest.

## Publisher’s note

All claims expressed in this article are solely those of the authors and do not necessarily represent those of their affiliated organizations, or those of the publisher, the editors and the reviewers. Any product that may be evaluated in this article, or claim that may be made by its manufacturer, is not guaranteed or endorsed by the publisher.

## Abbreviations

ALM, acral lentiginous melanoma; CCK-8, Cell Counting Kit-8; HCC, hepatocellular carcinoma; IHC, immunohistochemical; MM, malignant melanoma; MMPs, metalloproteinases; PCDH9, protocadherin-9; RAC1, Ras-related C3 botulinum toxin substrate 1.
