# Supplementary material for: PCDH9 suppresses melanoma proliferation and cell migration
Source: Front Oncol. 2022 Nov 14;12:903554. doi: 10.3389/fonc.2022.903554 (PMC9703089; doi:10.3389/fonc.2022.903554)
Supplement: Supplementary file 1 [file DataSheet_1.zip › Table 1.pdf]

Supplementary

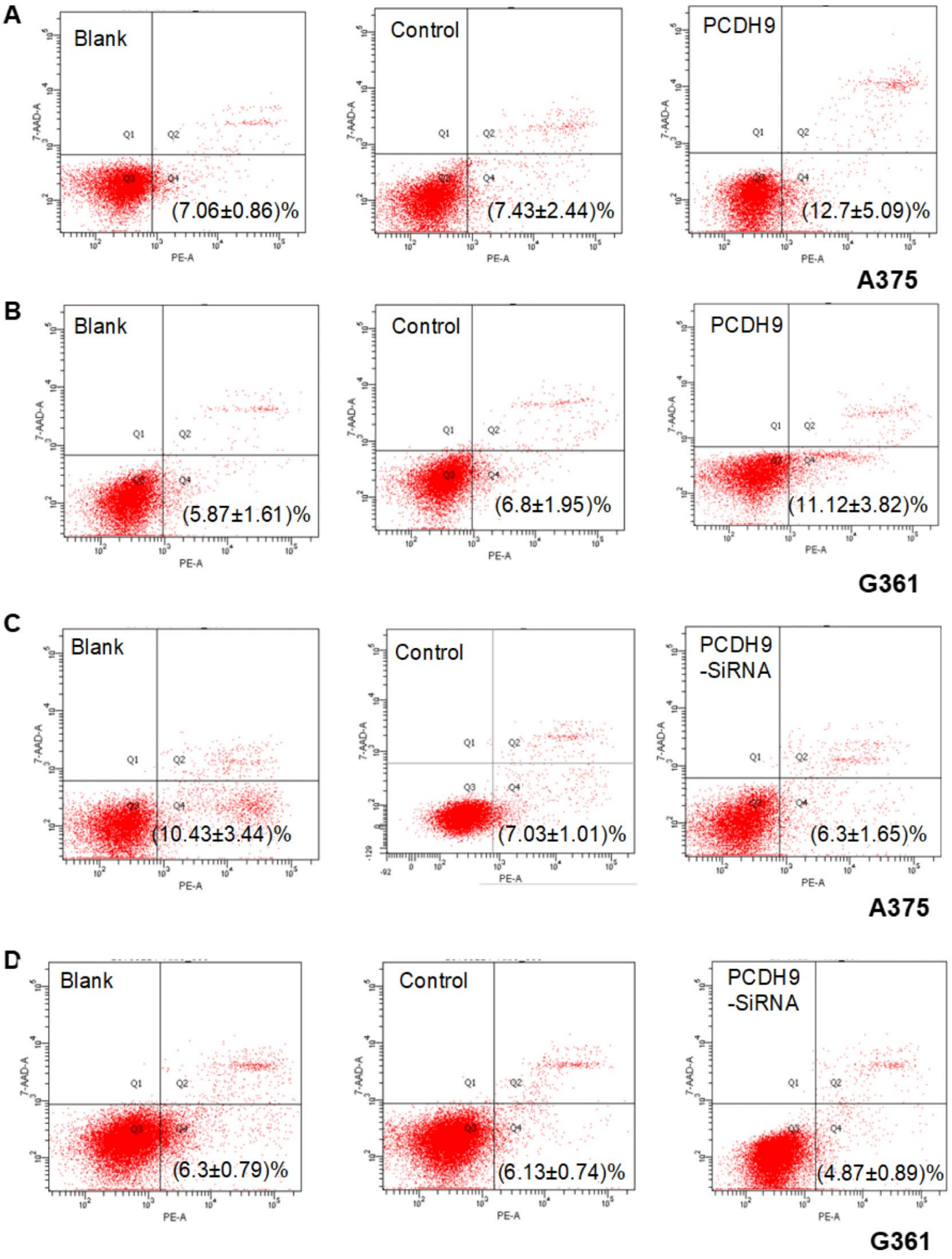

S1. The images of cell cycle were analyzed by flow cytometry: the images displayed the cell percentage of melanoma cells affected by overexpressed PCDH9 in different cell period time in A375 (**A**) and G361 (**B**) cell lines. The images of flow cytometry

displayed the cell percentage of melanoma cells affected by PCDH9 interference in different cell period time in A375 (**C**) and G361 (**D**) cell lines.

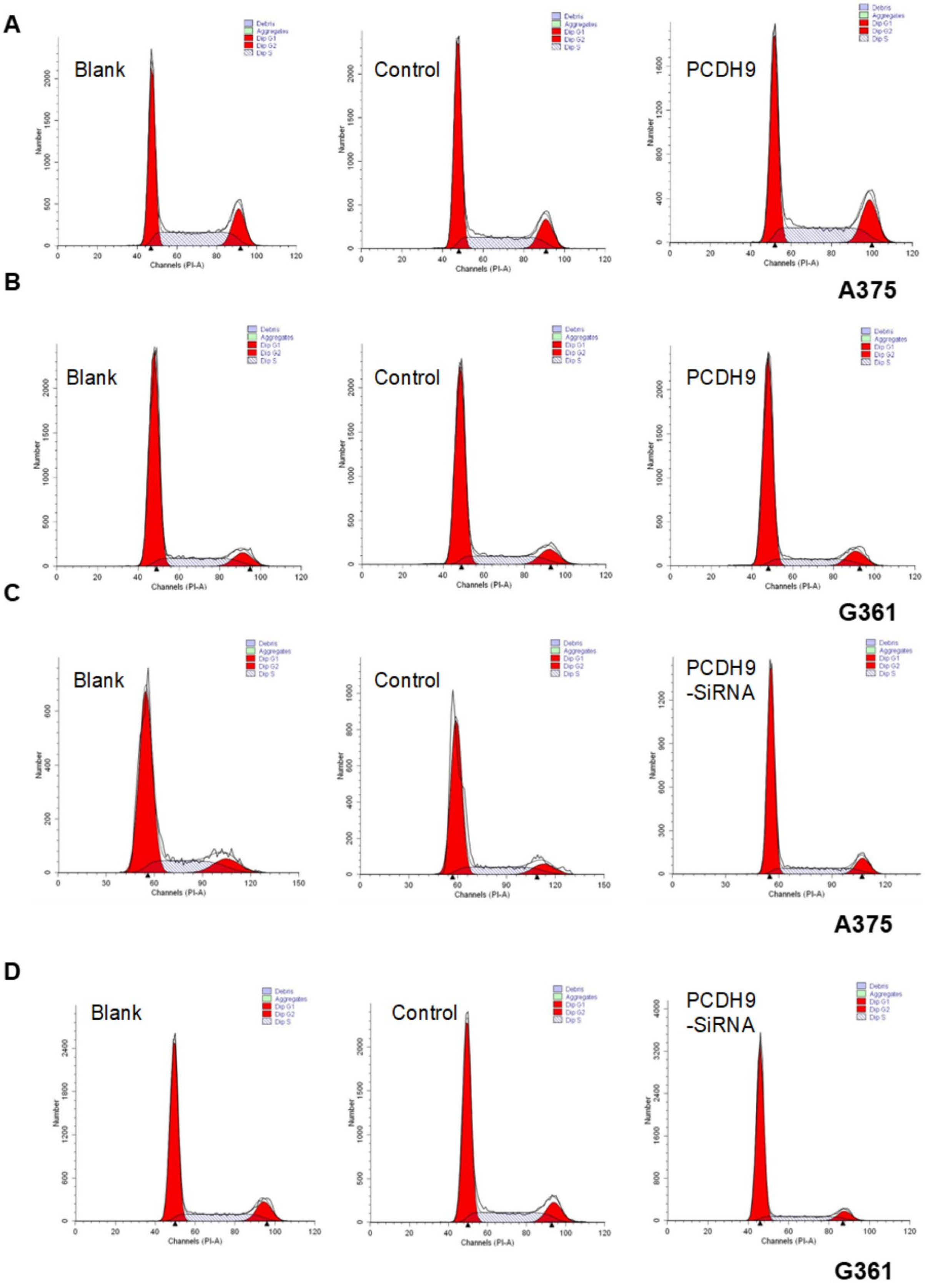

S2. The images of cell cycle were analyzed by flow cytometry: the images displayed the cell percentage of melanoma cells affected by overexpressed PCDH9 in different cell period time in A375 (**A**) and G361 (**B**) cell lines. The images of flow cytometry displayed the cell percentage of melanoma cells affected by PCDH9 interference in different cell period time in A375 (**C**) and G361 (**D**) cell lines.

S3. Primers designed for the amplifications of selected genes and amplicon sizes.

Name	Sequences (5'→3')	Sizes (bp)
PCDH9	F: TCCCAACTCTGATGGGCCTTTGGG<br>R: GGCTCTGGTCAGGGTGTGCC	217
CCND1	F: AGGAGAACAACTCTGACAACCACAATC<br>R: GCTCTTGATCGTCCTCTGACCAATAC	93
MMP2	F: TTTGACGGTAAGGACGGACTC<br>R: TACTCCCCATCGGCGTTC<br>R: TCAGGGCGAGGACCATAGAG	146
RAC1	F: ACAAGCCGATTGCCGATGTGTTC<br>R: TGCCGCACCTCAGGATACCAC	97
GAPDH	F: TAAAAGCAGCCCTGGTGACC<br>R: CCACATCGCTCAGACACCAT	88
