# Supplementary figures and images for: PCDH9 suppresses melanoma proliferation and cell migration
Source: Front Oncol. 2022 Nov 14;12:903554. doi: 10.3389/fonc.2022.903554 (PMC9703089; doi:10.3389/fonc.2022.903554)

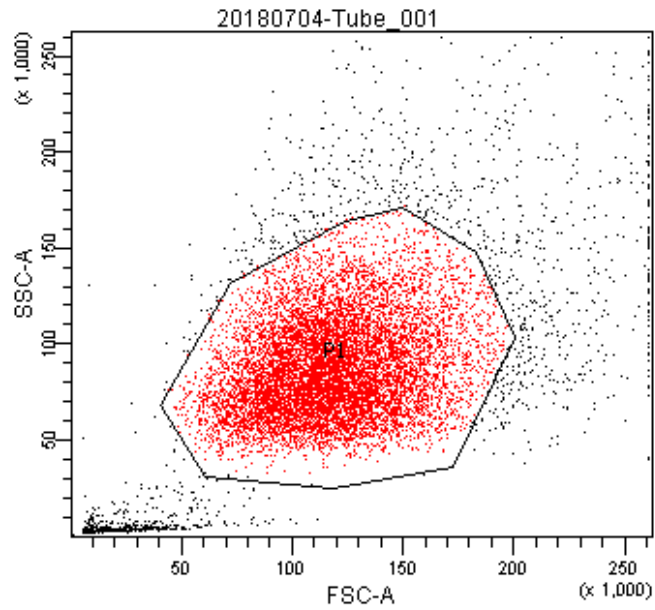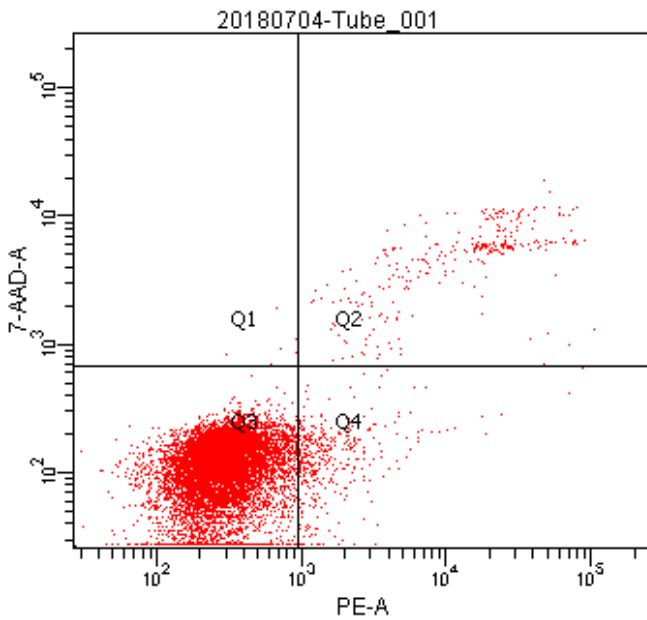

| Tube: Tube_001 |         |         |        |
|----------------|---------|---------|--------|
| Population     | #Events | %Parent | %Total |
| ■ All Events   | 12,037  | ###     | 100.0  |
| ■ P1           | 10,076  | 83.7    | 83.7   |
| □ Q1           | 5       | 0.0     | 0.0    |
| □ Q2           | 291     | 2.9     | 2.4    |
| □ Q3           | 9,378   | 93.1    | 77.9   |
| □ Q4           | 402     | 4.0     | 3.3    |

Supplement: Supplementary file 1 [file DataSheet_1.zip › Data Sheet 1.PDF]

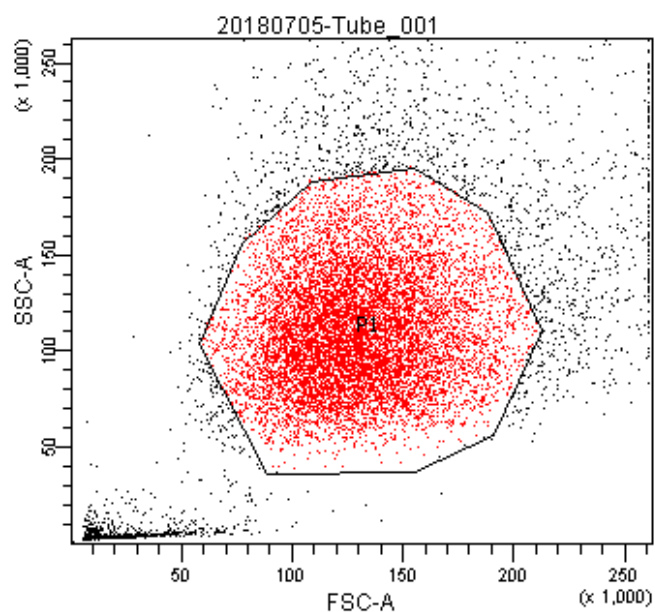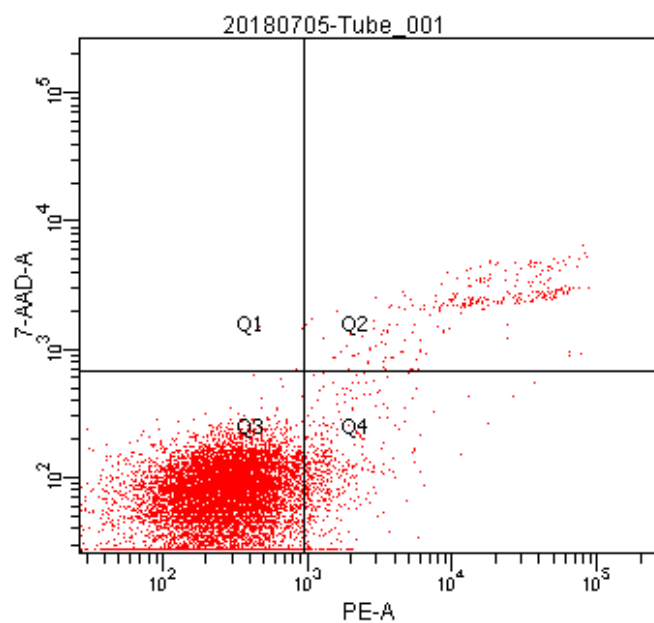

| Tube: Tube_001 |         |         |        |
|----------------|---------|---------|--------|
| Population     | #Events | %Parent | %Total |
| ■ All Events   | 13,208  | ####    | 100.0  |
| ■ P1           | 10,372  | 78.5    | 78.5   |
| □ Q1           | 2       | 0.0     | 0.0    |
| □ Q2           | 256     | 2.5     | 1.9    |
| □ Q3           | 9,652   | 93.1    | 73.1   |
| □ Q4           | 462     | 4.5     | 3.5    |

Supplement: Supplementary file 1 [file DataSheet_1.zip › Data Sheet 10.PDF]

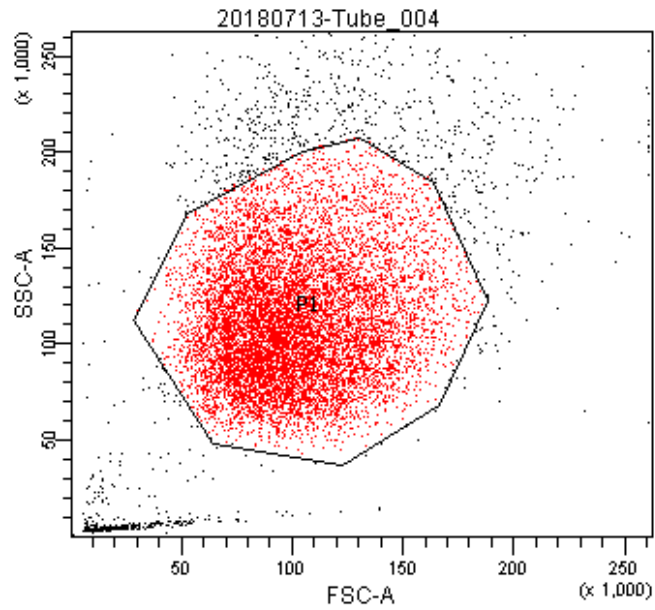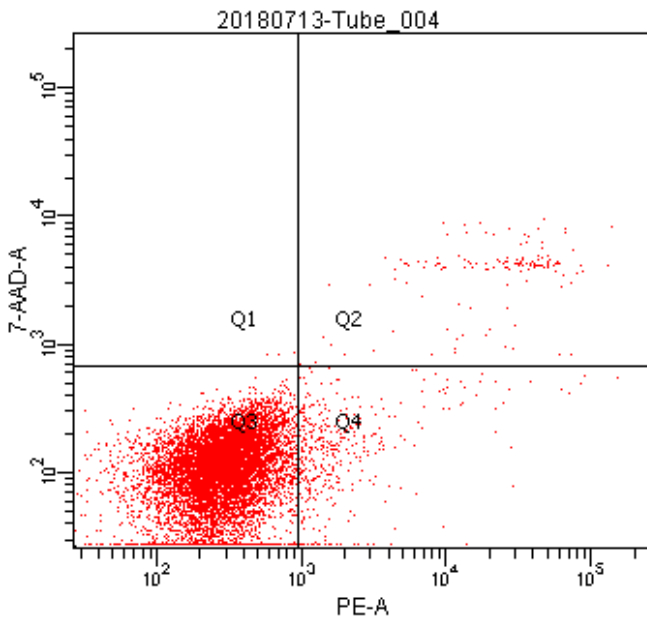

| Tube: Tube_004 |         |         |        |
|----------------|---------|---------|--------|
| Population     | #Events | %Parent | %Total |
| ■ All Events   | 11,439  | ###     | 100.0  |
| ■ P1           | 10,000  | 87.4    | 87.4   |
| □ Q1           | 3       | 0.0     | 0.0    |
| □ Q2           | 136     | 1.4     | 1.2    |
| □ Q3           | 9,459   | 94.6    | 82.7   |
| □ Q4           | 402     | 4.0     | 3.5    |

Supplement: Supplementary file 1 [file DataSheet_1.zip › Data Sheet 11.PDF]

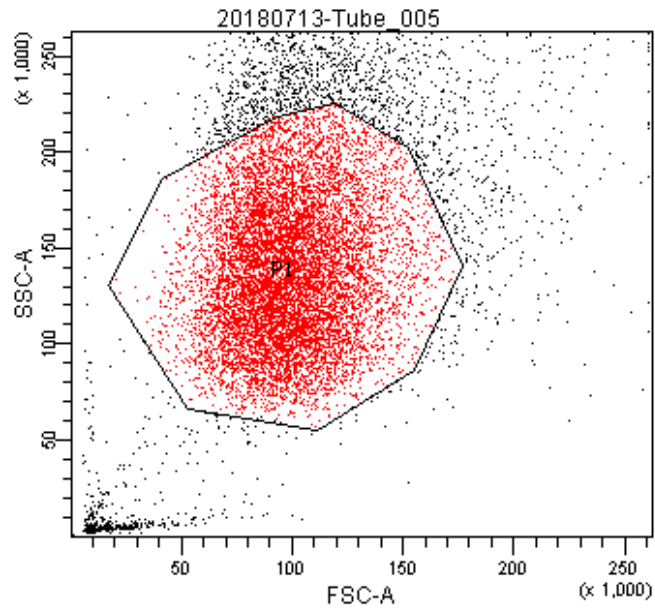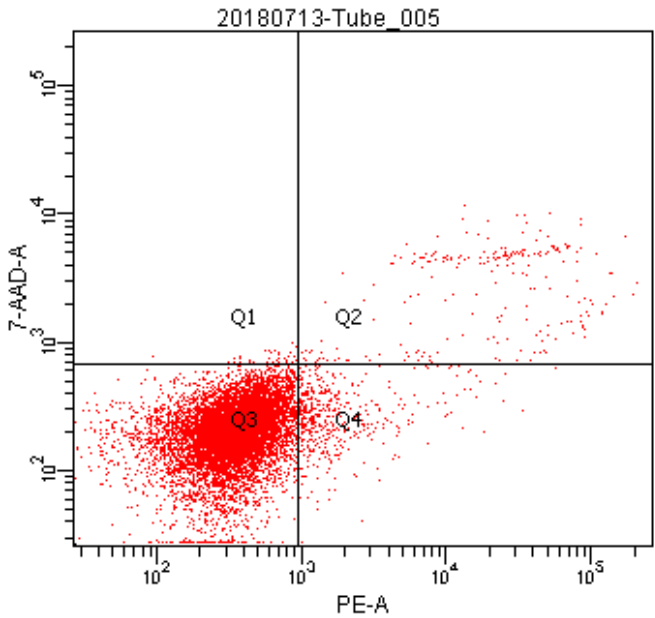

|                |         |         |        |
|----------------|---------|---------|--------|
| Tube: Tube_005 |         |         |        |
| Population     | #Events | %Parent | %Total |
| ■ All Events   | 13,710  | ###     | 100.0  |
| ■ P1           | 10,229  | 74.6    | 74.6   |
| ☒ Q1           | 23      | 0.2     | 0.2    |
| ☒ Q2           | 232     | 2.3     | 1.7    |
| ☒ Q3           | 9,501   | 92.9    | 69.3   |
| ☒ Q4           | 473     | 4.6     | 3.5    |

Supplement: Supplementary file 1 [file DataSheet_1.zip › Data Sheet 12.PDF]

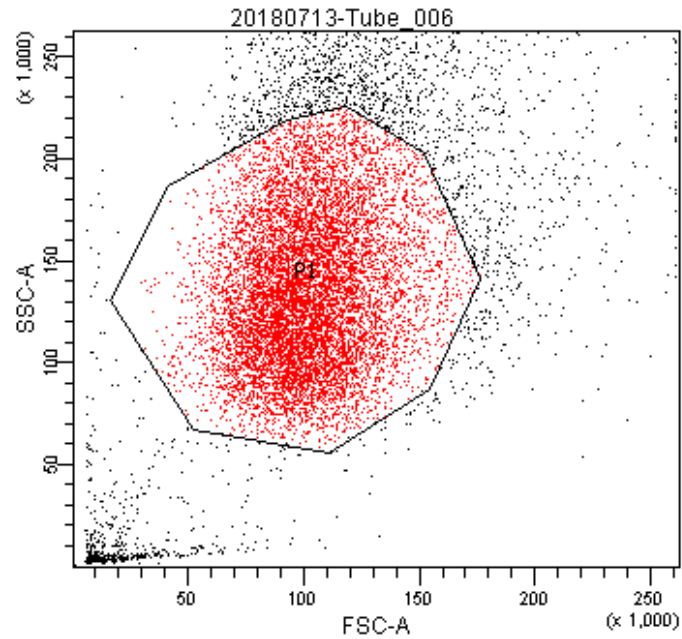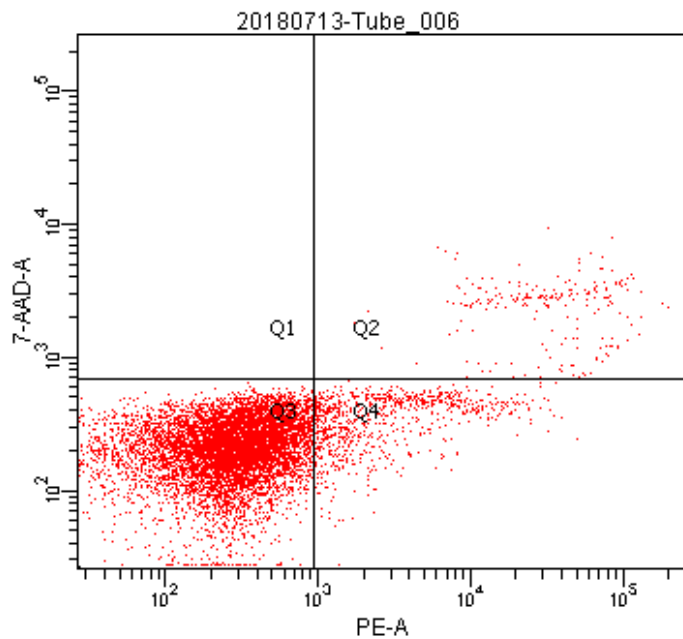

|                |         |         |        |
|----------------|---------|---------|--------|
| Tube: Tube_006 |         |         |        |
| Population     |         |         |        |
| ■ All Events   | #Events | %Parent | %Total |
| ■ P1           | 13,505  | ###     | 100.0  |
| ▣ Q1           | 10,000  | 74.0    | 74.0   |
| ▣ Q2           | 0       | 0.0     | 0.0    |
| ▣ Q3           | 211     | 2.1     | 1.6    |
| ▣ Q4           | 8,956   | 89.6    | 66.3   |
|                | 833     | 8.3     | 6.2    |

Supplement: Supplementary file 1 [file DataSheet_1.zip › Data Sheet 13.PDF]

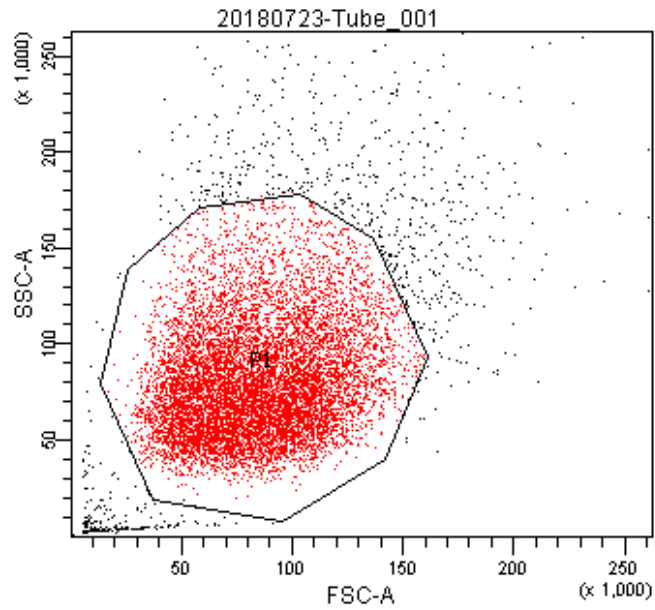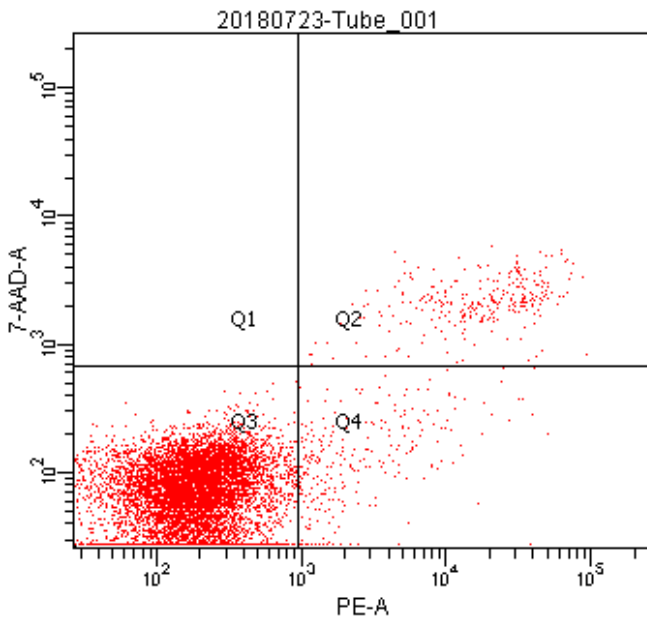

| Tube: Tube_001 |         |         |        |
|----------------|---------|---------|--------|
| Population     | #Events | %Parent | %Total |
| ■ All Events   | 10,972  | ####    | 100.0  |
| ■ P1           | 10,000  | 91.1    | 91.1   |
| ☒ Q1           | 0       | 0.0     | 0.0    |
| ☒ Q2           | 279     | 2.8     | 2.5    |
| ☒ Q3           | 9,447   | 94.5    | 86.1   |
| ☒ Q4           | 274     | 2.7     | 2.5    |

Supplement: Supplementary file 1 [file DataSheet_1.zip › Data Sheet 14.PDF]

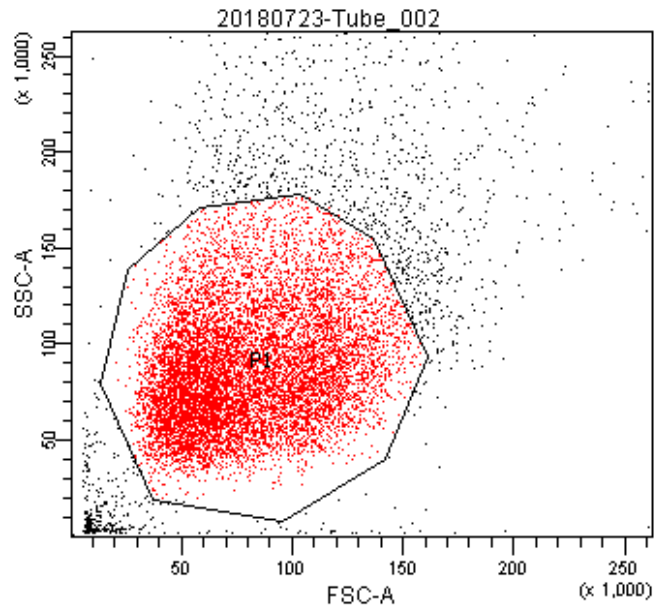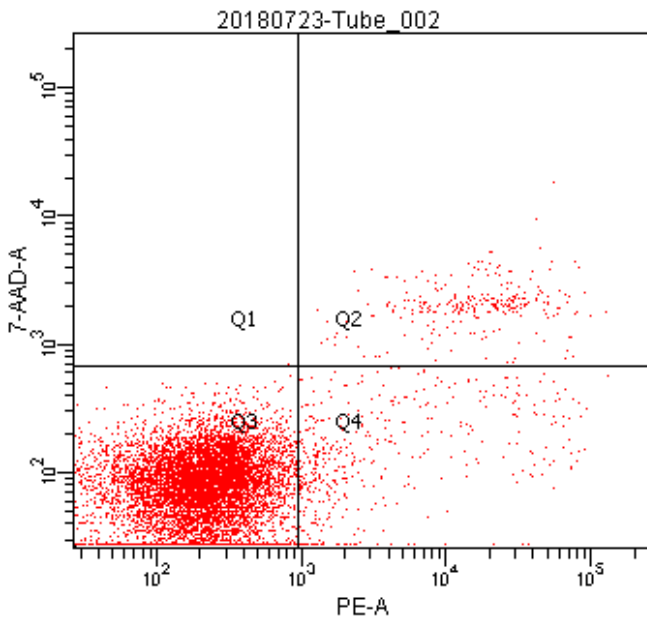

| Tube: Tube_002 |         |         |        |
|----------------|---------|---------|--------|
| Population     | #Events | %Parent | %Total |
| ■ All Events   | 11,605  | ###     | 100.0  |
| ■ P1           | 10,000  | 86.2    | 86.2   |
| □ Q1           | 0       | 0.0     | 0.0    |
| □ Q2           | 270     | 2.7     | 2.3    |
| □ Q3           | 9,283   | 92.8    | 80.0   |
| □ Q4           | 447     | 4.5     | 3.9    |

Supplement: Supplementary file 1 [file DataSheet_1.zip › Data Sheet 15.PDF]

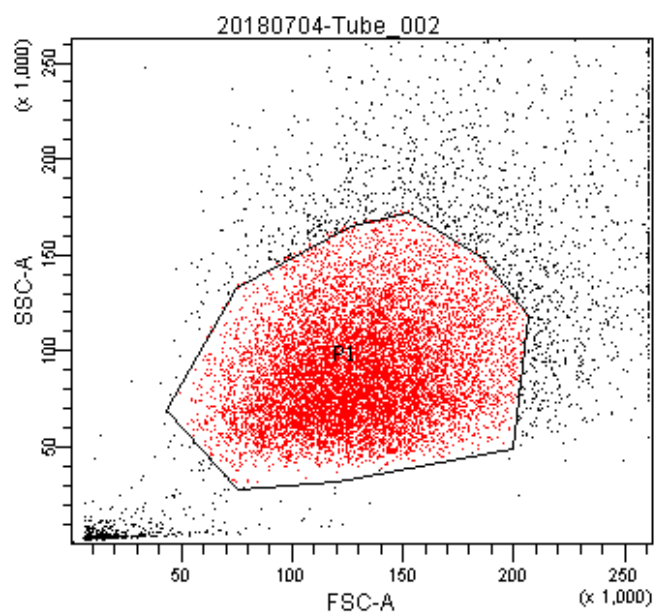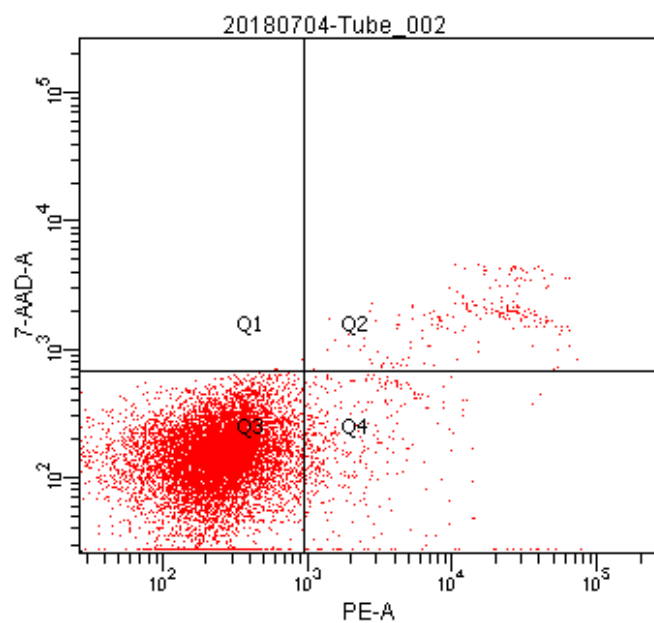

| Tube: Tube_002 |         |         |        |
|----------------|---------|---------|--------|
| Population     | #Events | %Parent | %Total |
| ■ All Events   | 12,849  | ###     | 100.0  |
| ■ P1           | 10,410  | 81.0    | 81.0   |
| □ Q1           | 1       | 0.0     | 0.0    |
| □ Q2           | 202     | 1.9     | 1.6    |
| □ Q3           | 9,820   | 94.3    | 76.4   |
| □ Q4           | 387     | 3.7     | 3.0    |

Supplement: Supplementary file 1 [file DataSheet_1.zip › Data Sheet 2.PDF]

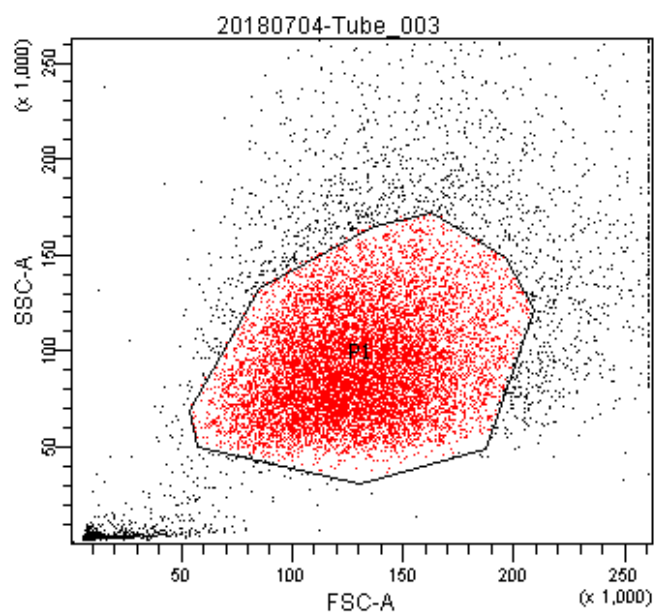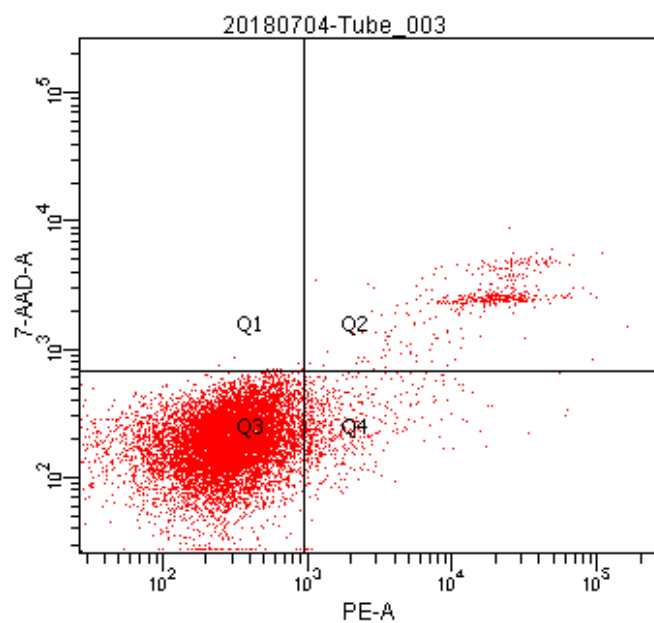

| Tube: Tube_003 |         |         |        |
|----------------|---------|---------|--------|
| Population     | #Events | %Parent | %Total |
| ■ All Events   | 13,001  | ####    | 100.0  |
| ■ P1           | 10,192  | 78.4    | 78.4   |
| □ Q1           | 3       | 0.0     | 0.0    |
| □ Q2           | 401     | 3.9     | 3.1    |
| □ Q3           | 9,266   | 90.9    | 71.3   |
| □ Q4           | 522     | 5.1     | 4.0    |

Supplement: Supplementary file 1 [file DataSheet_1.zip › Data Sheet 3.PDF]

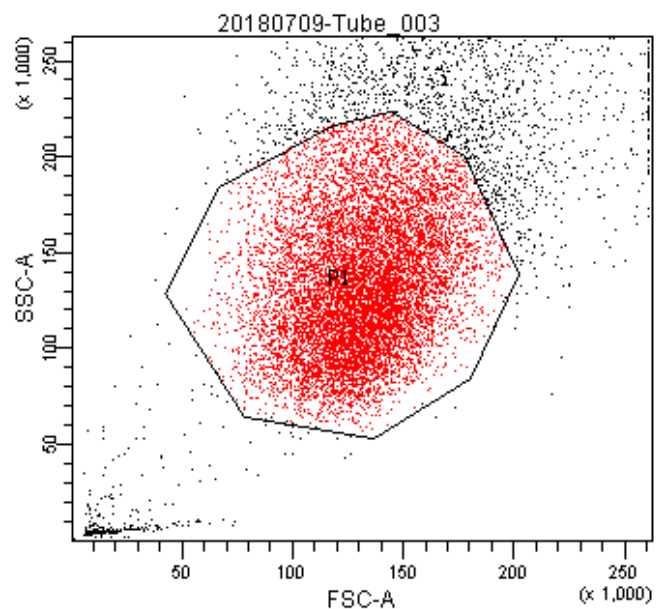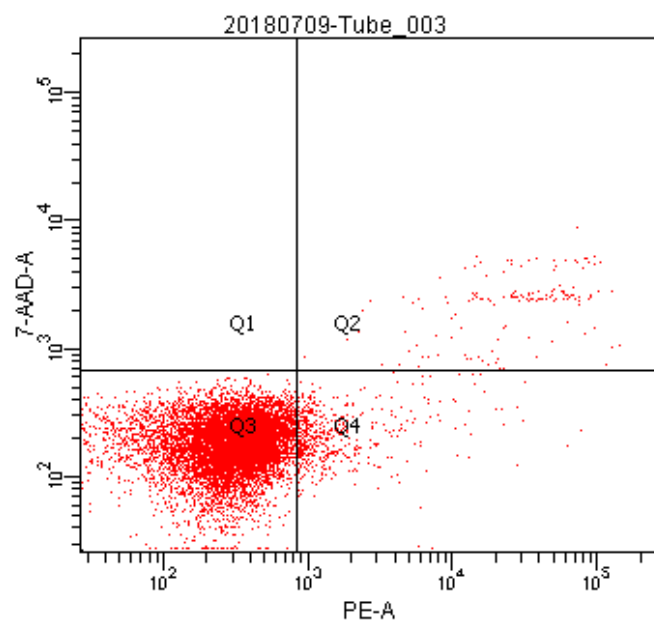

| Tube: Tube_003 |         |         |        |
|----------------|---------|---------|--------|
| Population     | #Events | %Parent | %Total |
| ■ All Events   | 13,372  | ####    | 100.0  |
| ■ P1           | 10,580  | 79.1    | 79.1   |
| □ Q1           | 0       | 0.0     | 0.0    |
| □ Q2           | 142     | 1.3     | 1.1    |
| □ Q3           | 9,912   | 93.7    | 74.1   |
| □ Q4           | 526     | 5.0     | 3.9    |

Supplement: Supplementary file 1 [file DataSheet_1.zip › Data Sheet 4.PDF]

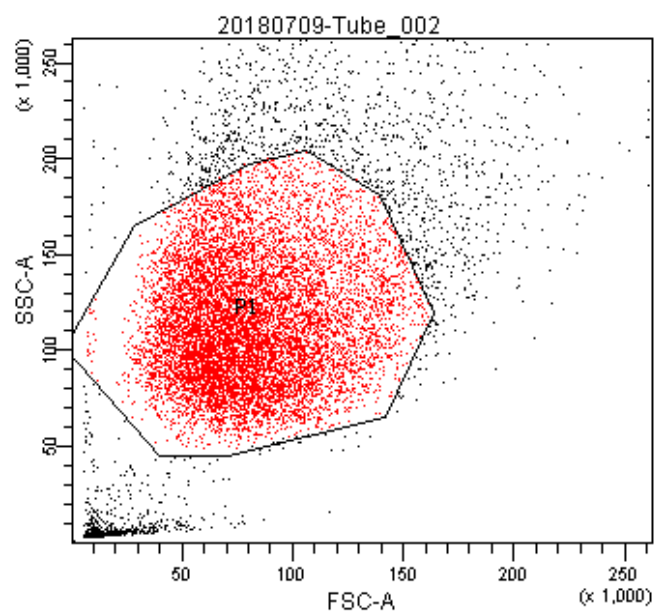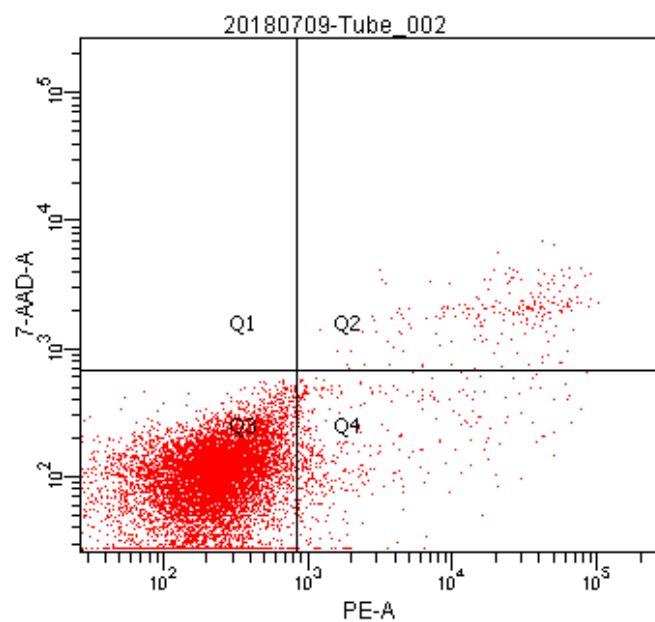

| Tube: Tube_002 |         |         |        |  |
|----------------|---------|---------|--------|--|
| Population     | #Events | %Parent | %Total |  |
| ■ All Events   | 12,691  | ####    | 100.0  |  |
| ■ P1           | 10,298  | 81.1    | 81.1   |  |
| □ Q1           | 0       | 0.0     | 0.0    |  |
| □ Q2           | 216     | 2.1     | 1.7    |  |
| □ Q3           | 9,634   | 93.6    | 75.9   |  |
| □ Q4           | 448     | 4.4     | 3.5    |  |

Supplement: Supplementary file 1 [file DataSheet_1.zip › Data Sheet 5.PDF]

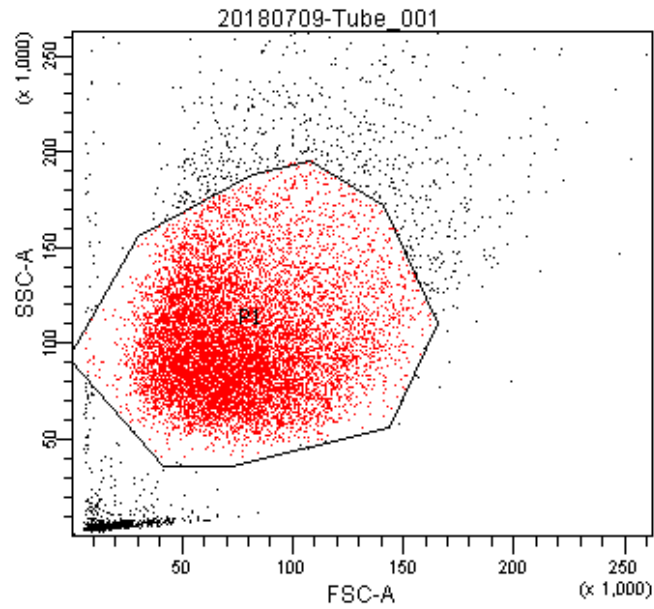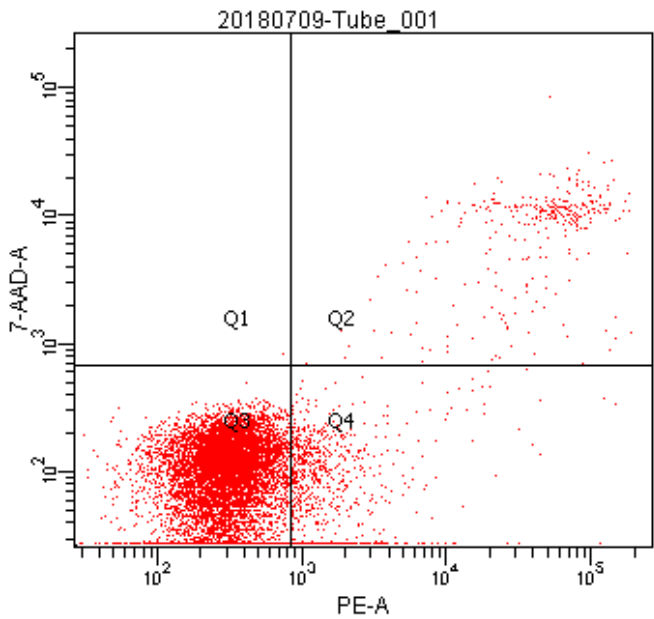

| Tube: Tube_001 |         |         |        |
|----------------|---------|---------|--------|
| Population     | #Events | %Parent | %Total |
| ■ All Events   | 12,160  | ####    | 100.0  |
| ■ P1           | 10,429  | 85.8    | 85.8   |
| □ Q1           | 1       | 0.0     | 0.0    |
| □ Q2           | 316     | 3.0     | 2.6    |
| □ Q3           | 9,315   | 89.3    | 76.6   |
| □ Q4           | 797     | 7.6     | 6.6    |

Supplement: Supplementary file 1 [file DataSheet_1.zip › Data Sheet 6.PDF]

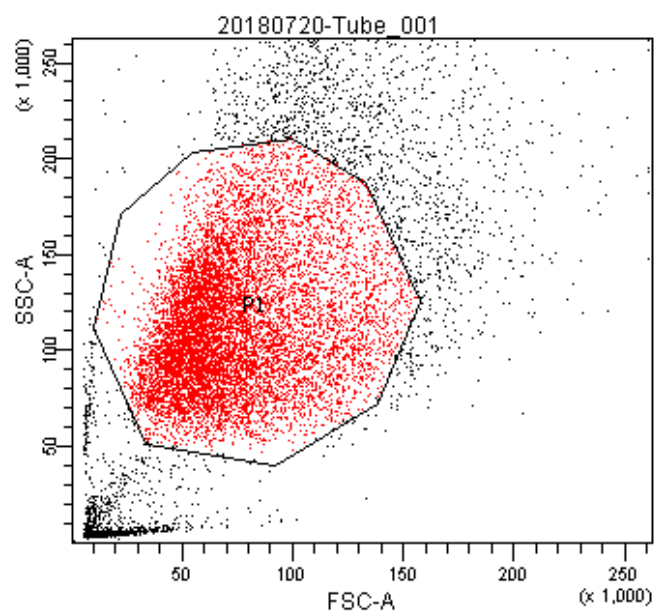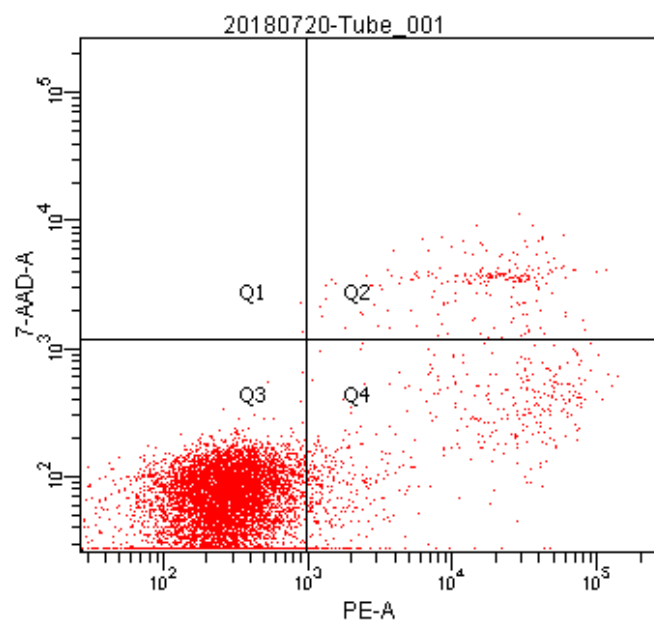

| Tube: Tube_001 |         |         |        |
|----------------|---------|---------|--------|
| Population     | #Events | %Parent | %Total |
| ■ All Events   | 13,877  | ####    | 100.0  |
| ■ P1           | 10,000  | 72.1    | 72.1   |
| □ Q1           | 2       | 0.0     | 0.0    |
| □ Q2           | 214     | 2.1     | 1.5    |
| □ Q3           | 9,189   | 91.9    | 66.2   |
| □ Q4           | 595     | 5.9     | 4.3    |

Supplement: Supplementary file 1 [file DataSheet_1.zip › Data Sheet 7.PDF]

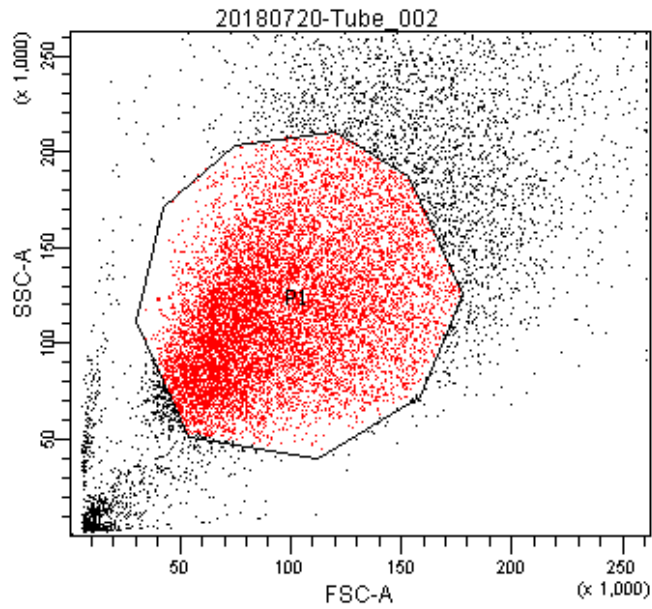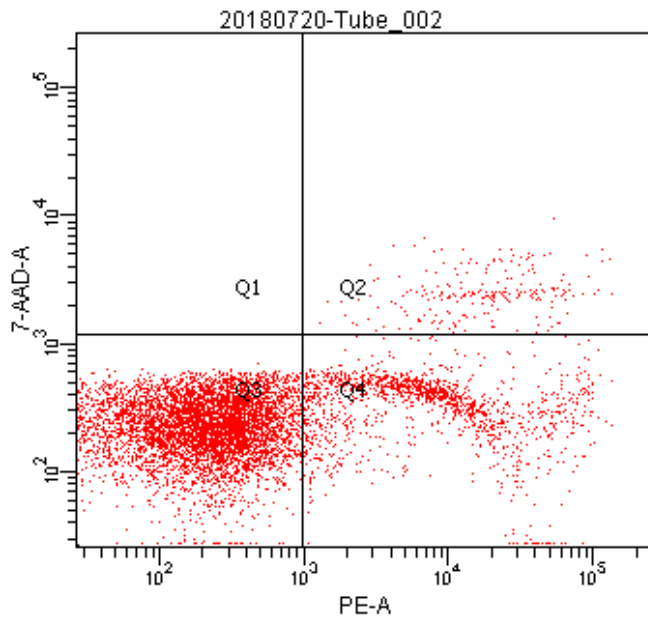

| Tube: Tube_002 |         |         |        |  |
|----------------|---------|---------|--------|--|
| Population     | #Events | %Parent | %Total |  |
| ■ All Events   | 15,700  | ####    | 100.0  |  |
| ■ P1           | 10,713  | 68.2    | 68.2   |  |
| □ Q1           | 0       | 0.0     | 0.0    |  |
| □ Q2           | 223     | 2.1     | 1.4    |  |
| □ Q3           | 9,008   | 84.1    | 57.4   |  |
| □ Q4           | 1,482   | 13.8    | 9.4    |  |

Supplement: Supplementary file 1 [file DataSheet_1.zip › Data Sheet 8.PDF]

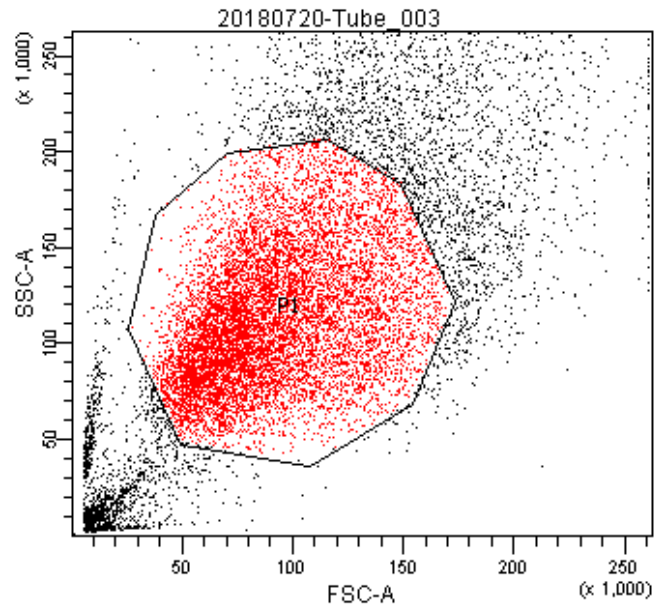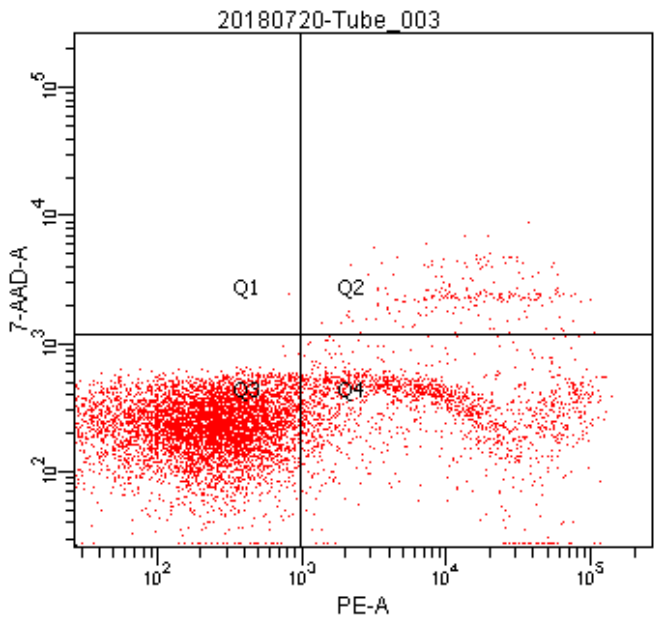

|                |         |         |        |
|----------------|---------|---------|--------|
| Tube: Tube_003 |         |         |        |
| Population     | #Events | %Parent | %Total |
| ■ All Events   | 15,120  | ###     | 100.0  |
| ■ P1           | 10,000  | 66.1    | 66.1   |
| □ Q1           | 1       | 0.0     | 0.0    |
| □ Q2           | 212     | 2.1     | 1.4    |
| □ Q3           | 8,148   | 81.5    | 53.9   |
| □ Q4           | 1,639   | 16.4    | 10.8   |

Supplement: Supplementary file 1 [file DataSheet_1.zip › Data Sheet 9.PDF]

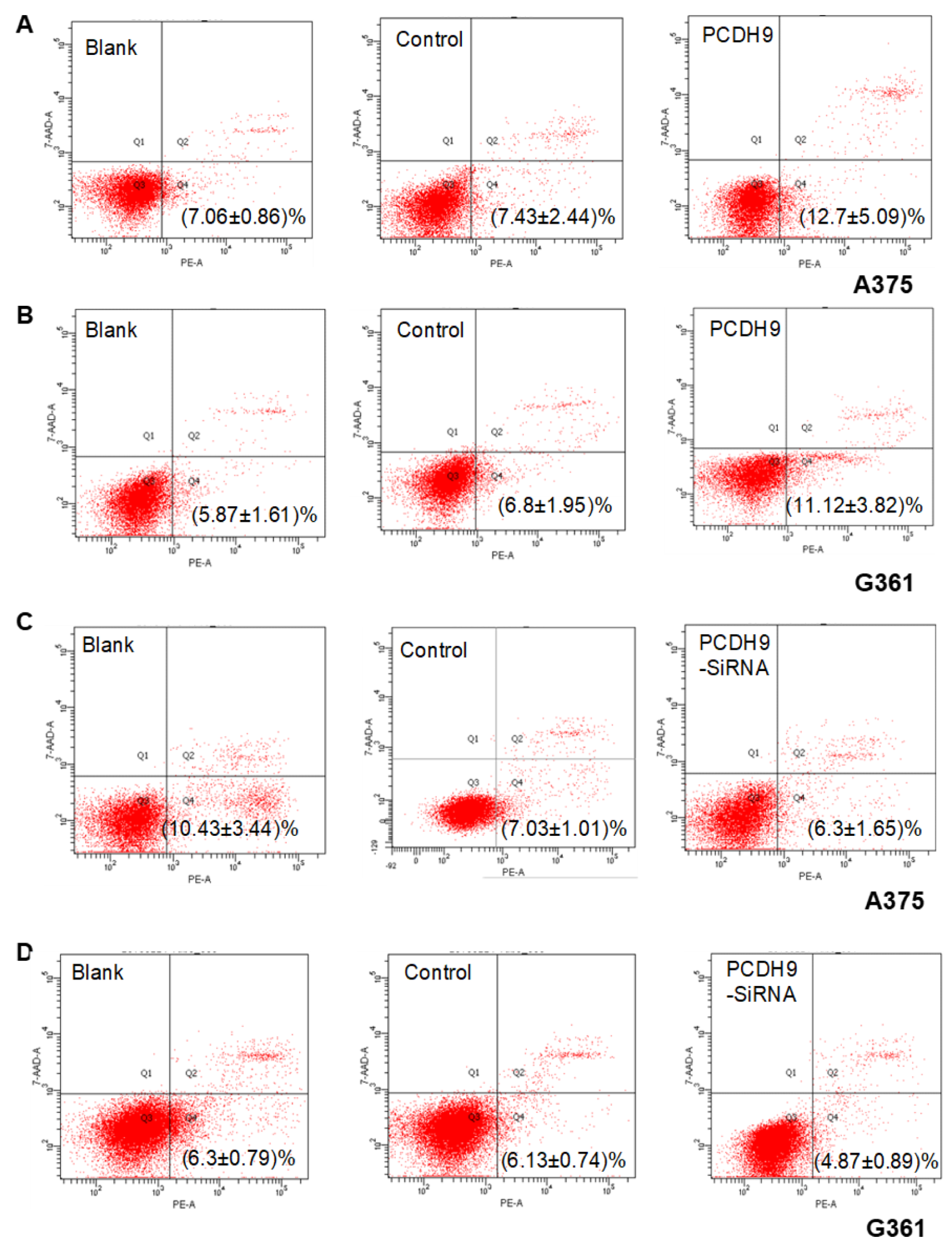

Supplement: Supplementary file 1 [file DataSheet_1.zip › Image 1.TIF]

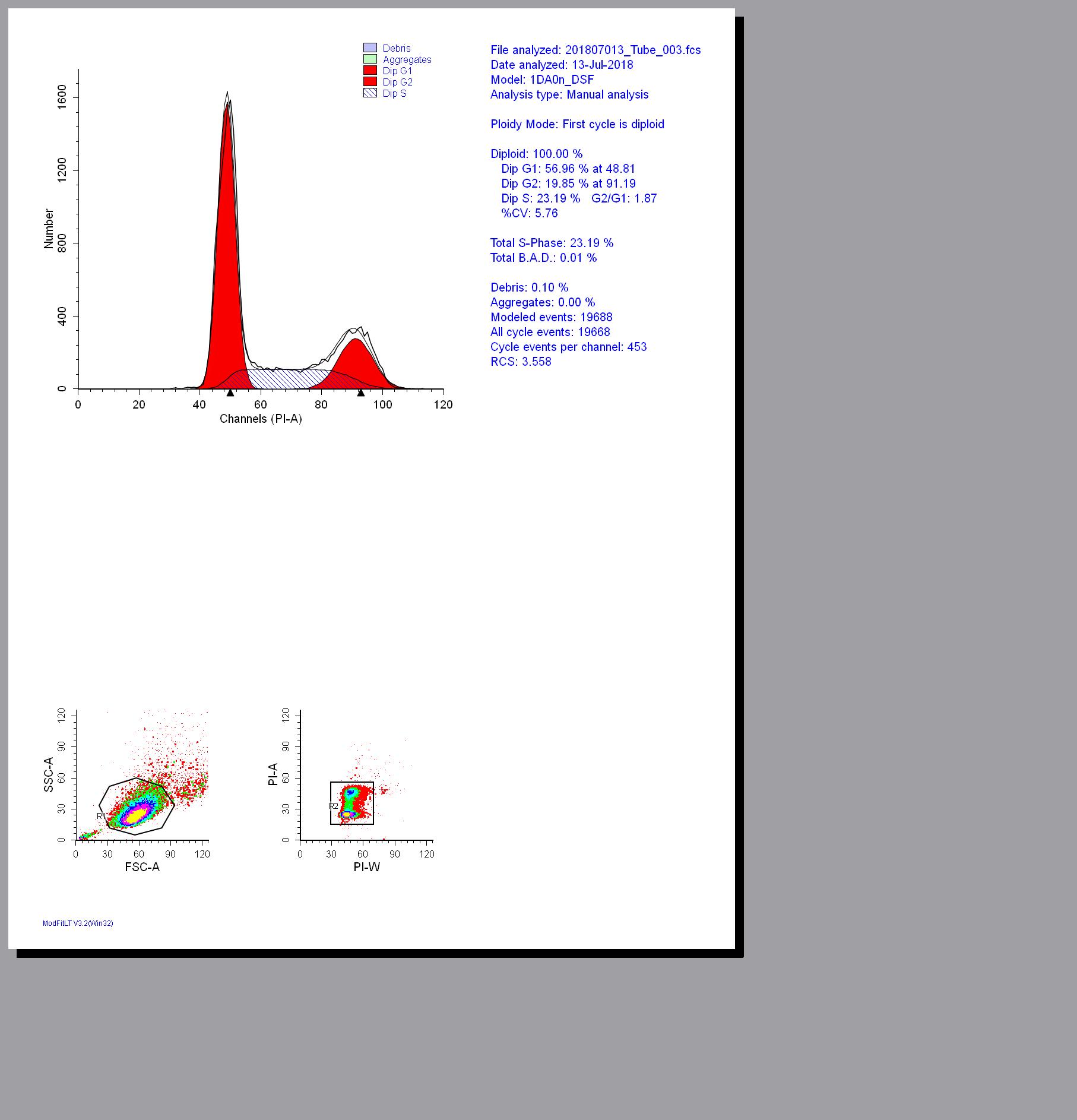

Supplement: Supplementary file 1 [file DataSheet_1.zip › Image 10.JPEG]

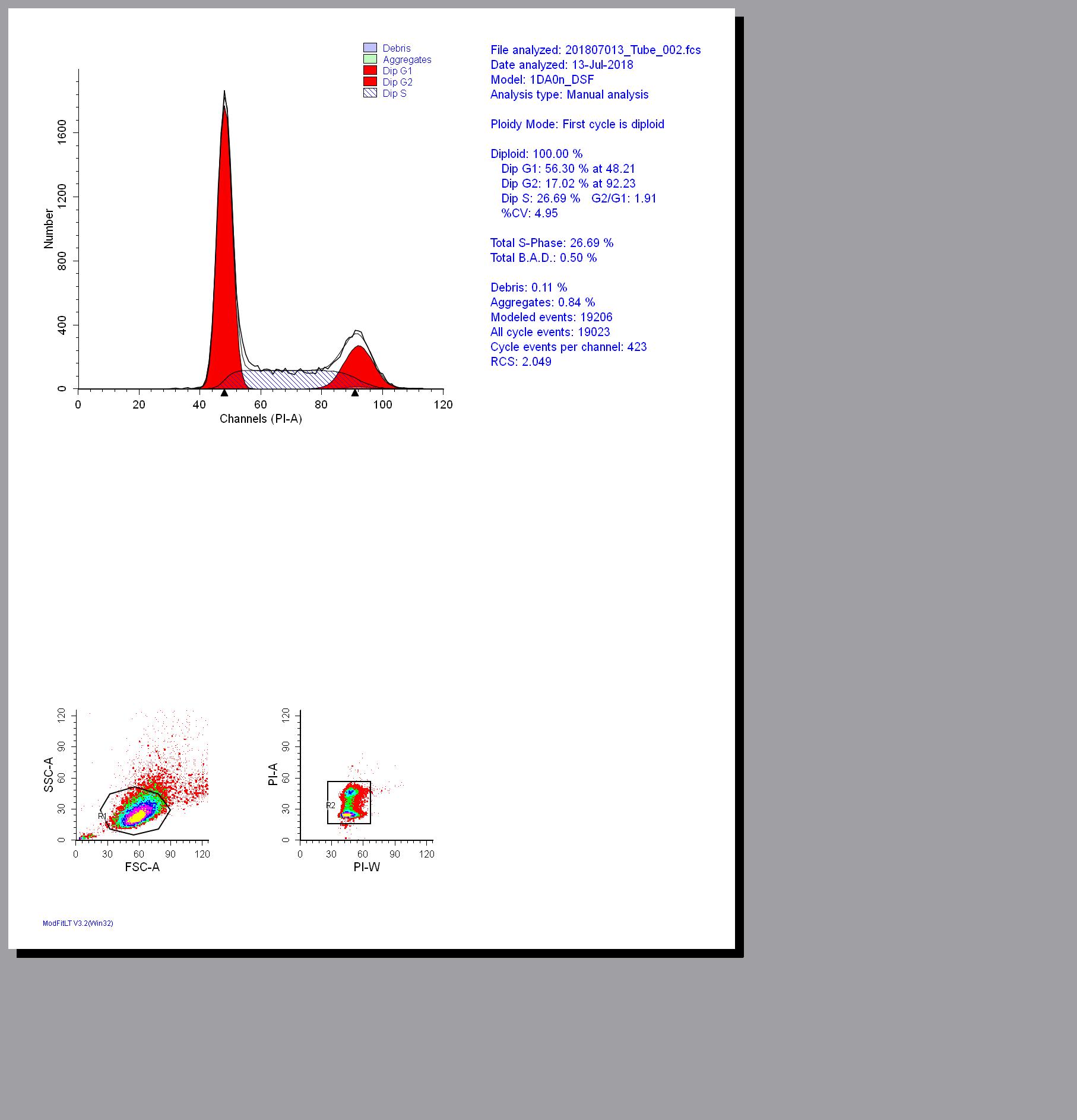

Supplement: Supplementary file 1 [file DataSheet_1.zip › Image 11.JPEG]

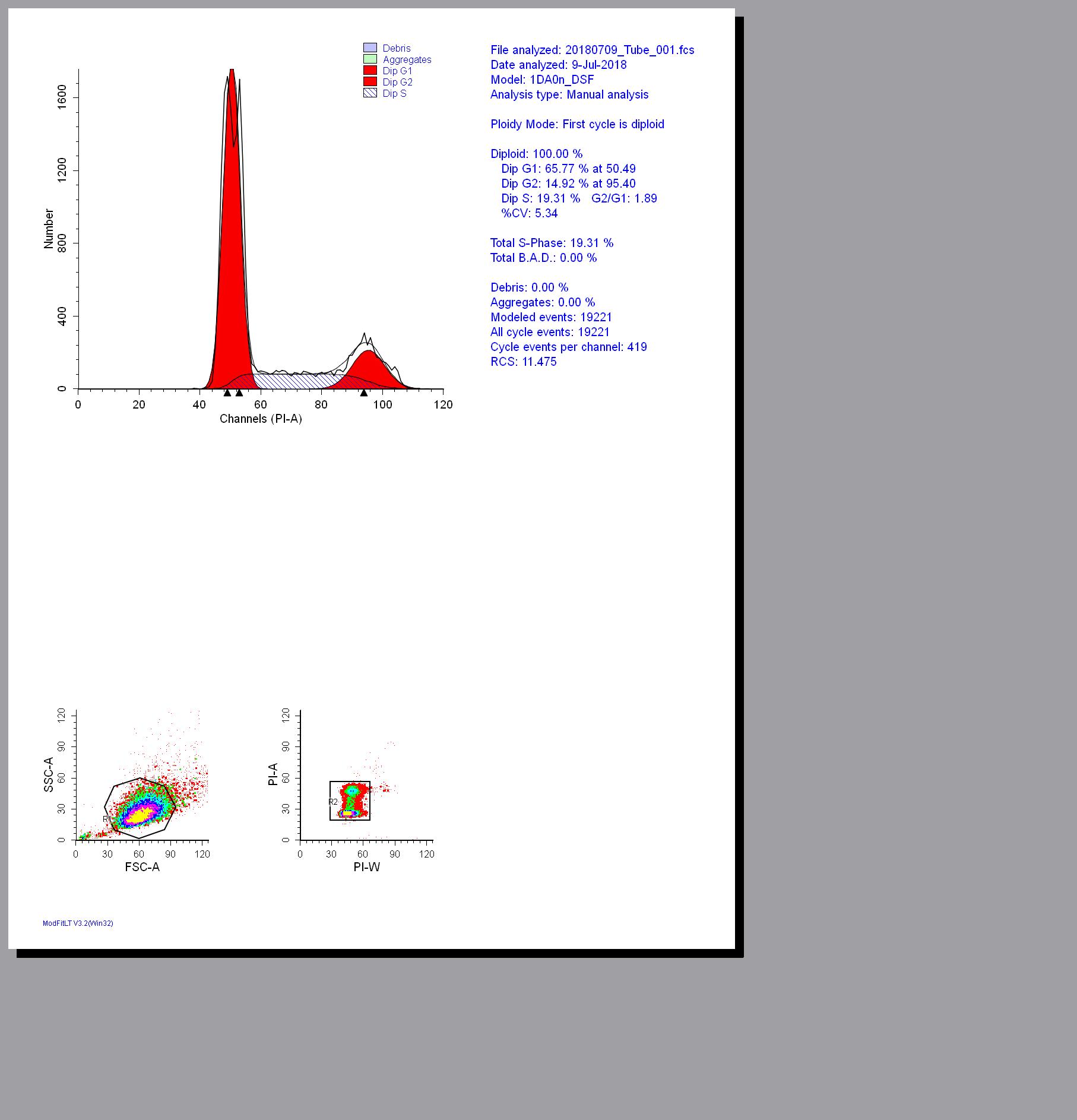

Supplement: Supplementary file 1 [file DataSheet_1.zip › Image 12.JPEG]

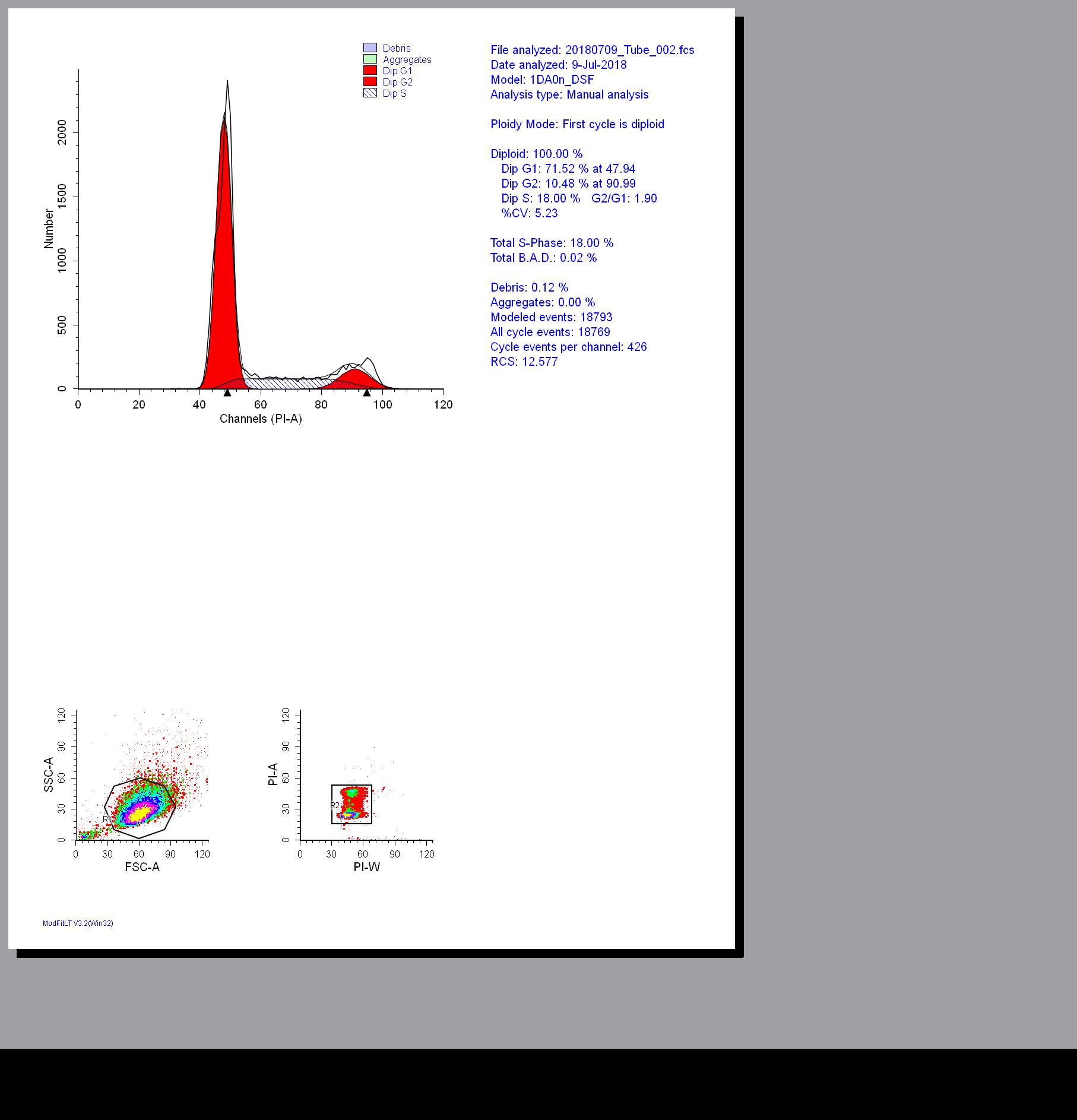

Supplement: Supplementary file 1 [file DataSheet_1.zip › Image 13.JPEG]

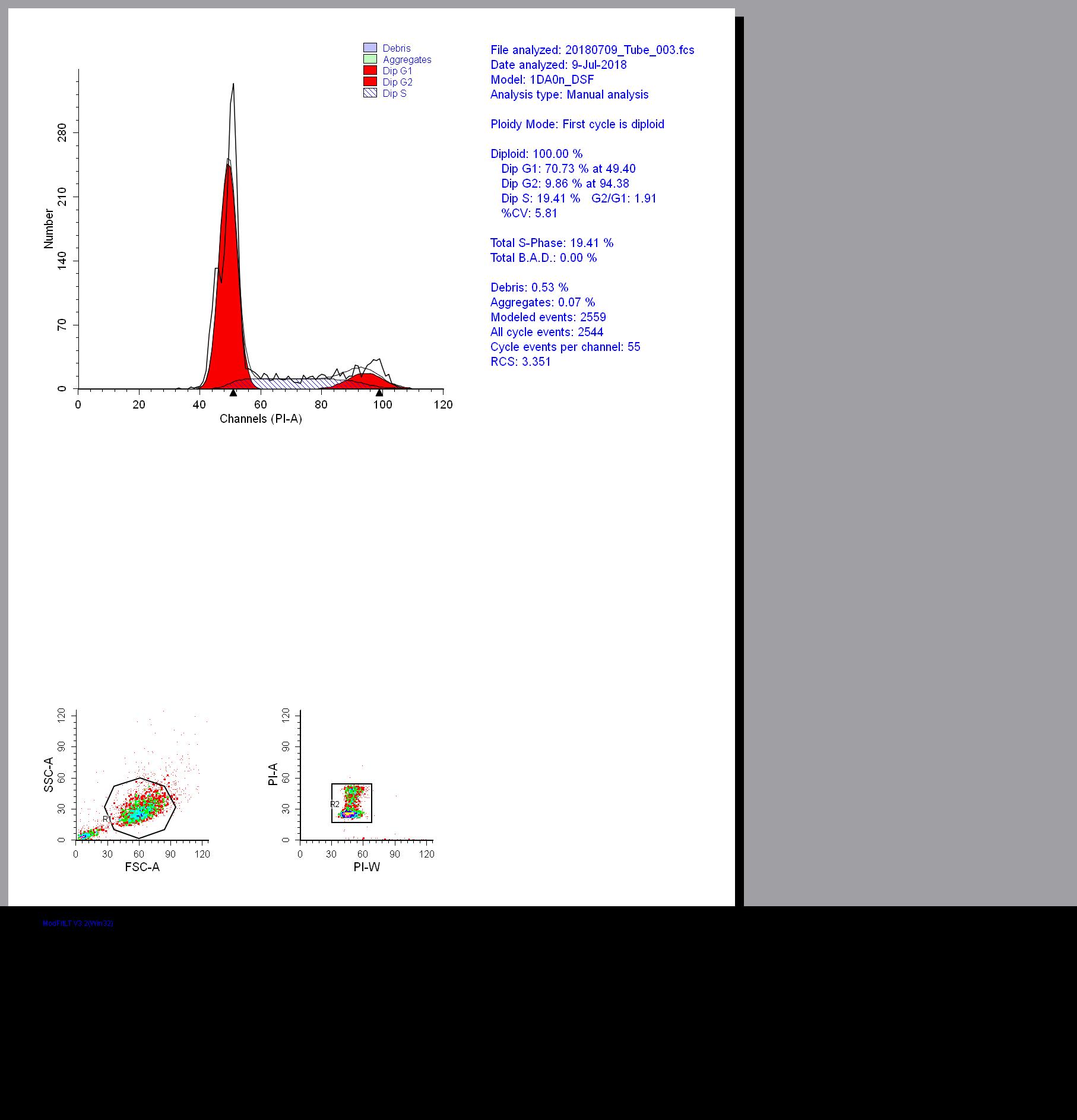

Supplement: Supplementary file 1 [file DataSheet_1.zip › Image 14.JPEG]

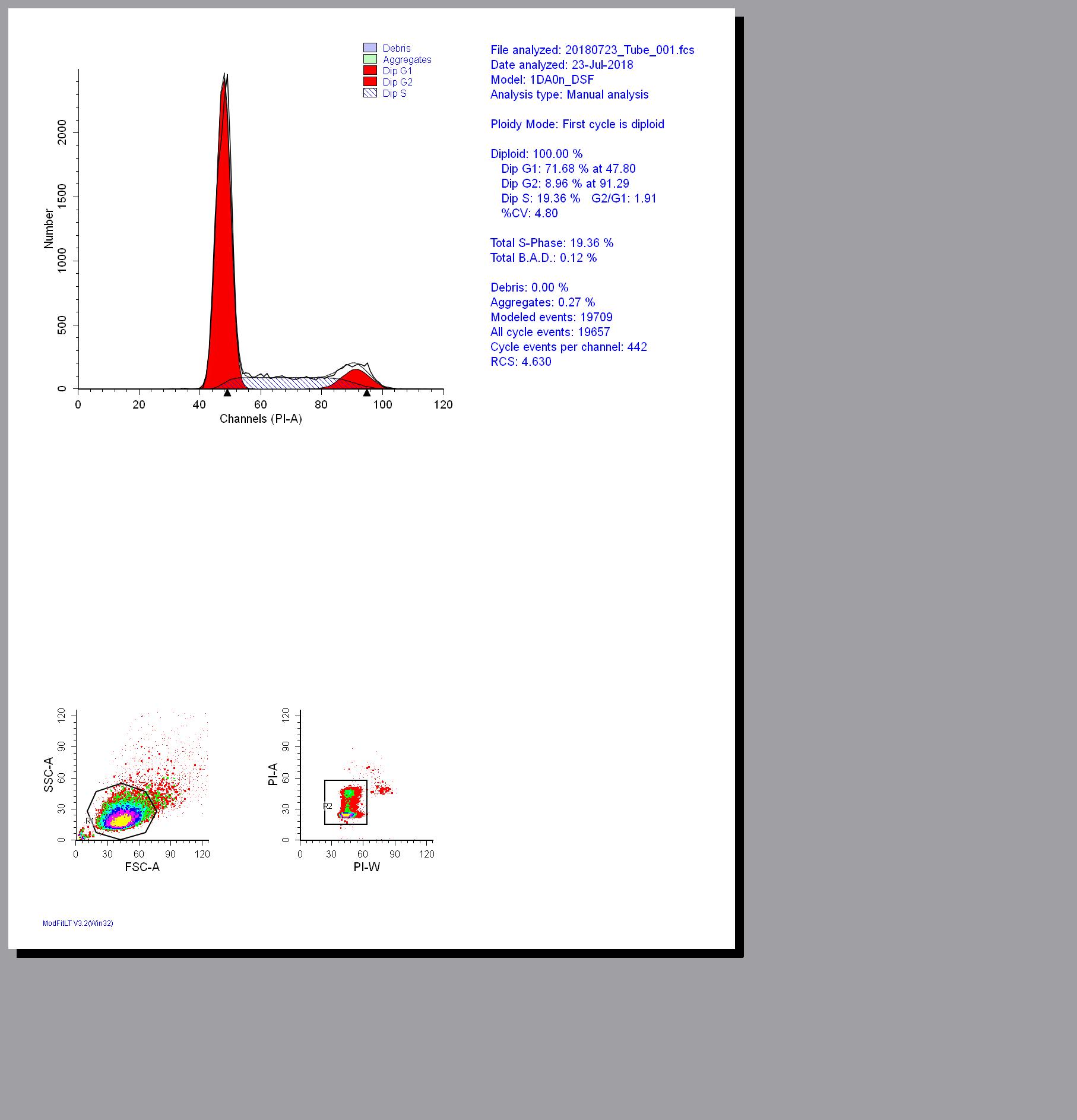

Supplement: Supplementary file 1 [file DataSheet_1.zip › Image 15.JPEG]

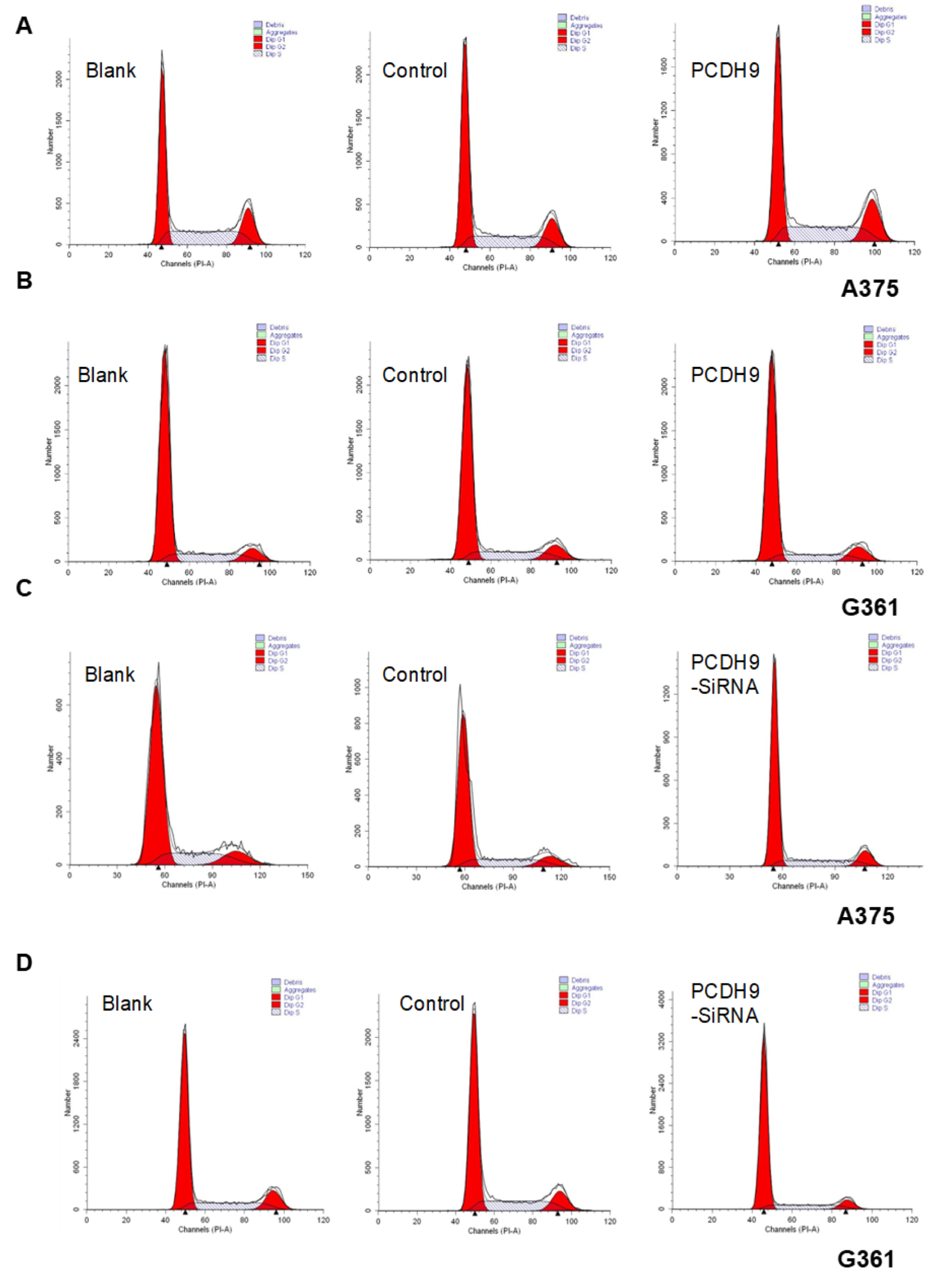

Supplement: Supplementary file 1 [file DataSheet_1.zip › Image 2.TIF]

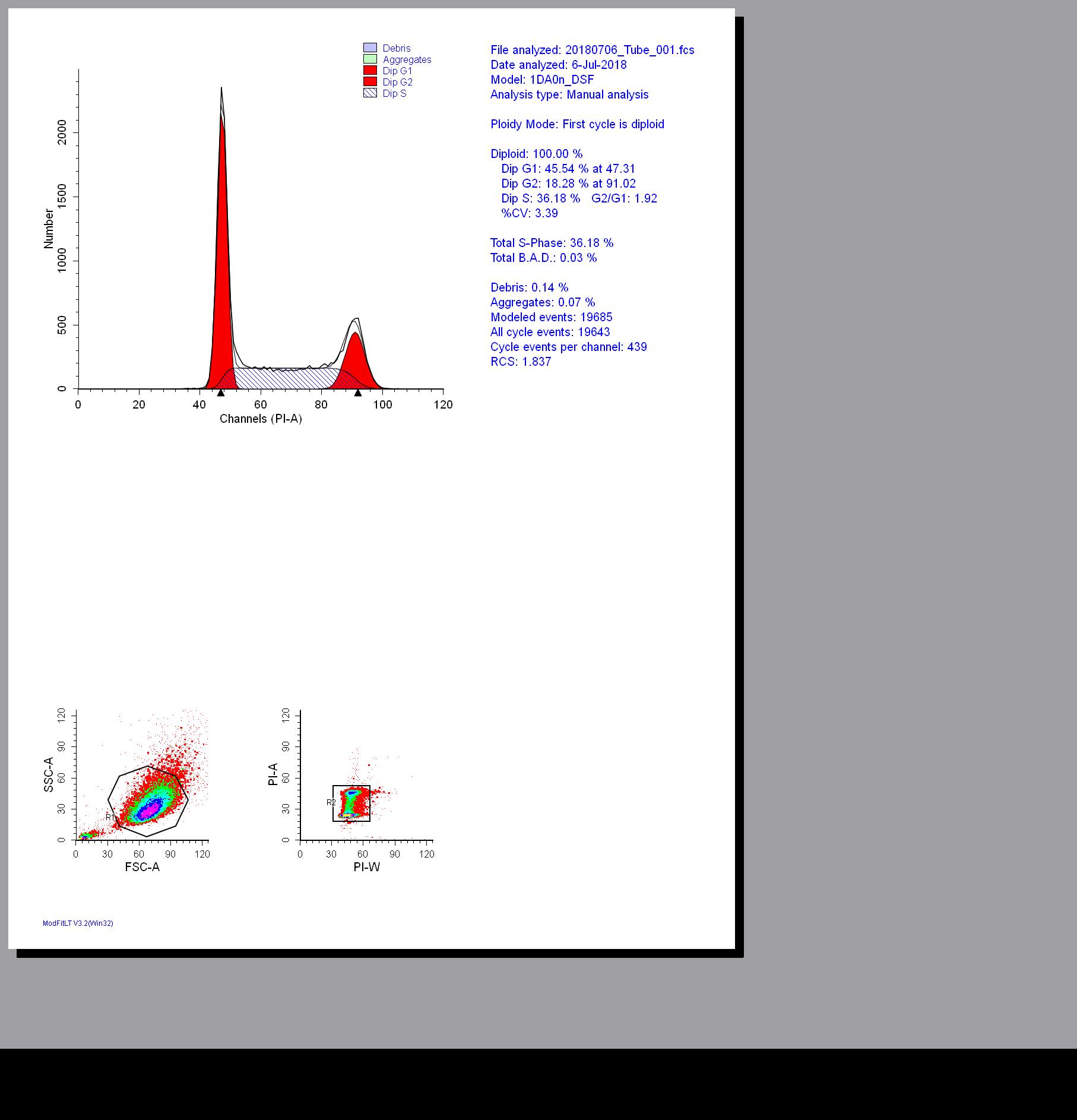

Supplement: Supplementary file 1 [file DataSheet_1.zip › Image 3.JPEG]

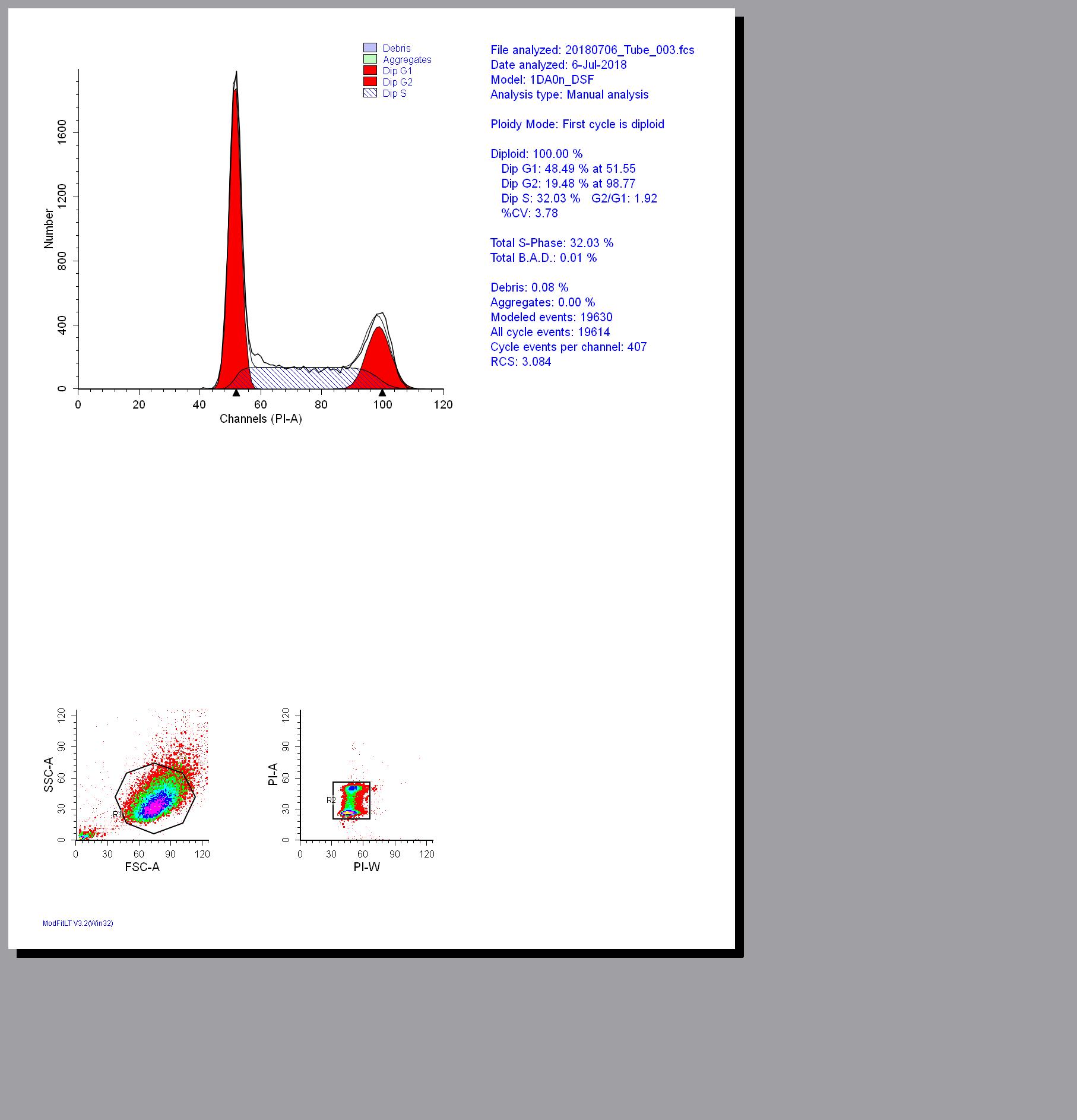

Supplement: Supplementary file 1 [file DataSheet_1.zip › Image 4.JPEG]

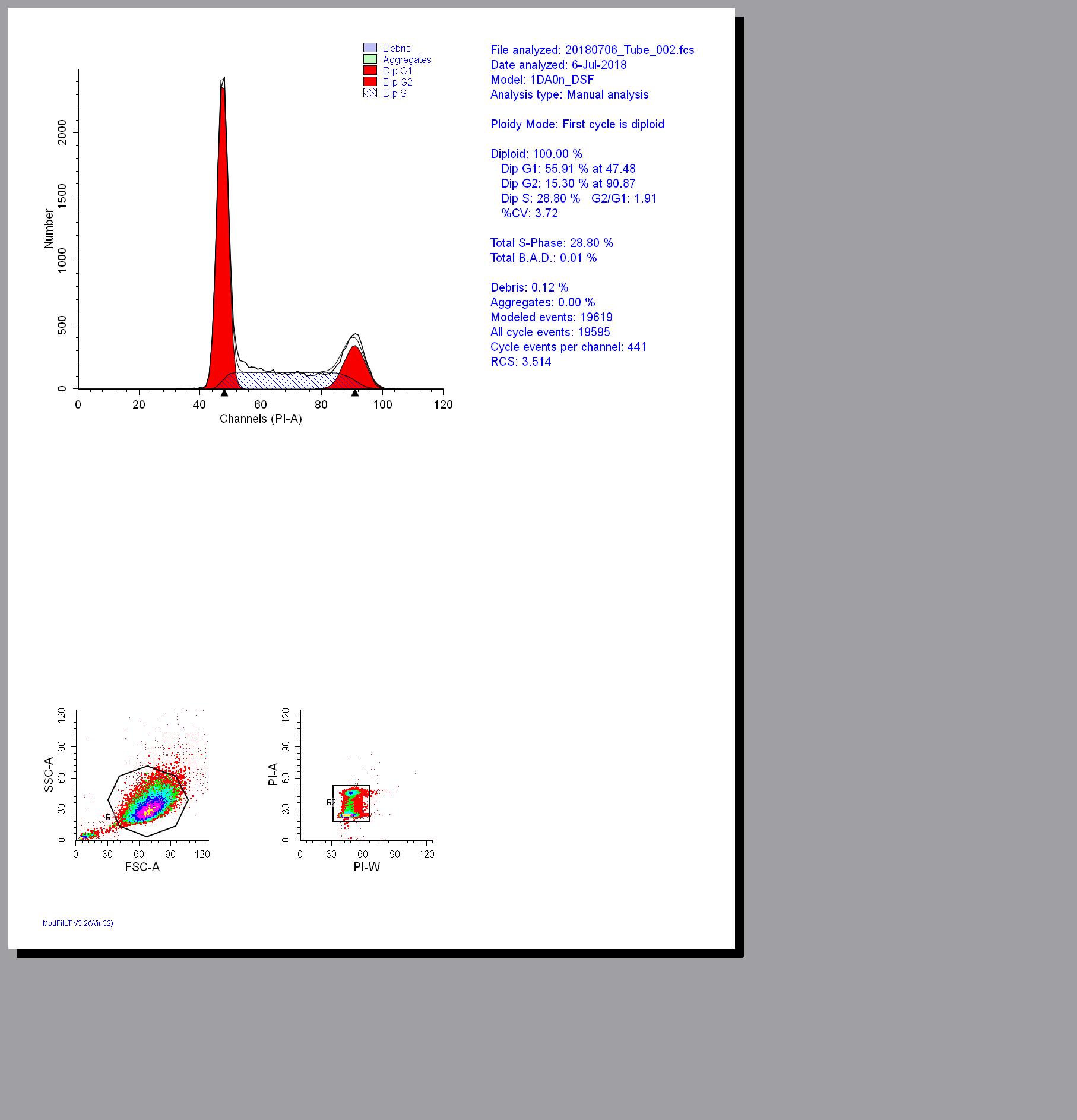

Supplement: Supplementary file 1 [file DataSheet_1.zip › Image 5.JPEG]

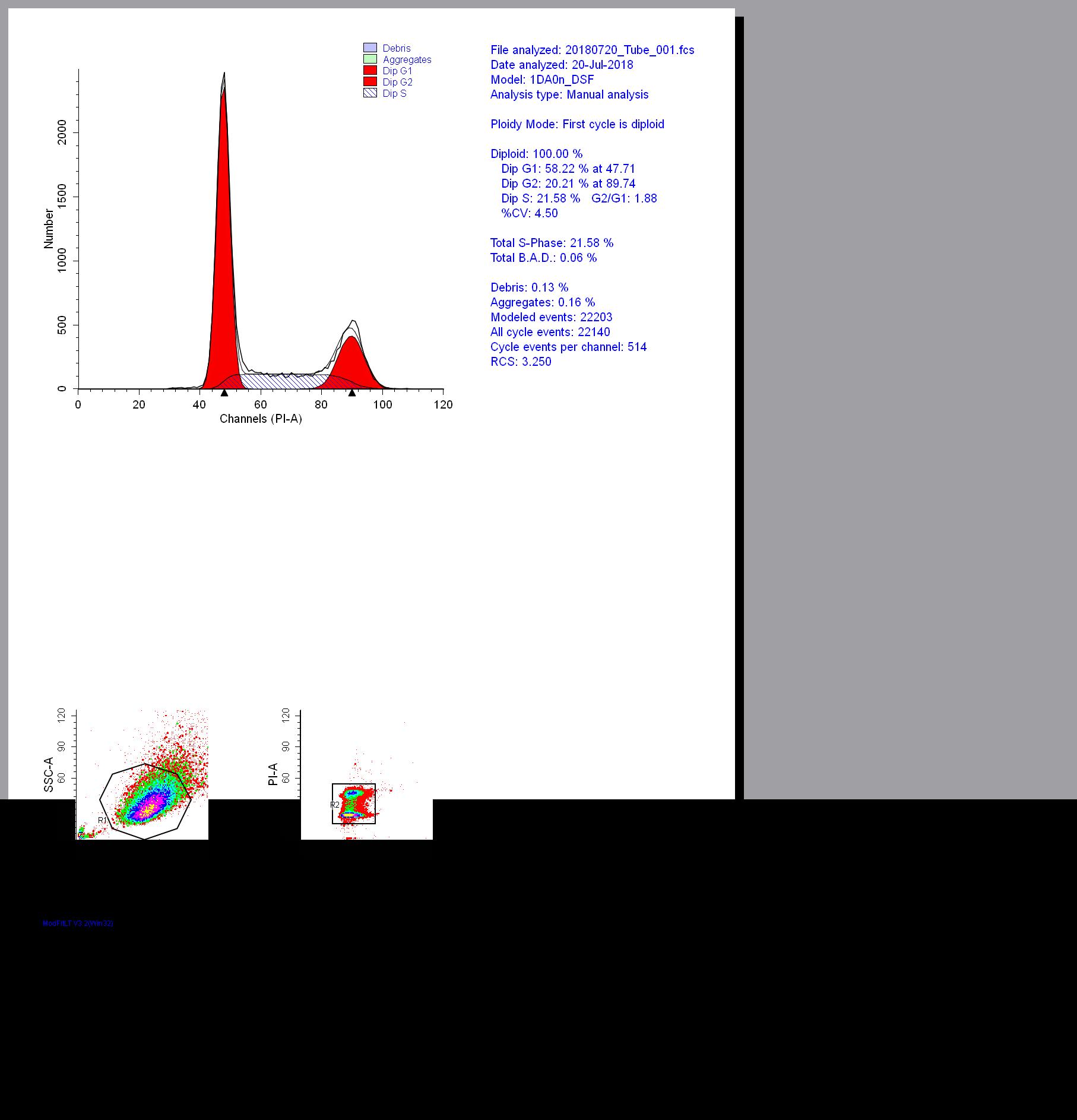

Supplement: Supplementary file 1 [file DataSheet_1.zip › Image 6.JPEG]

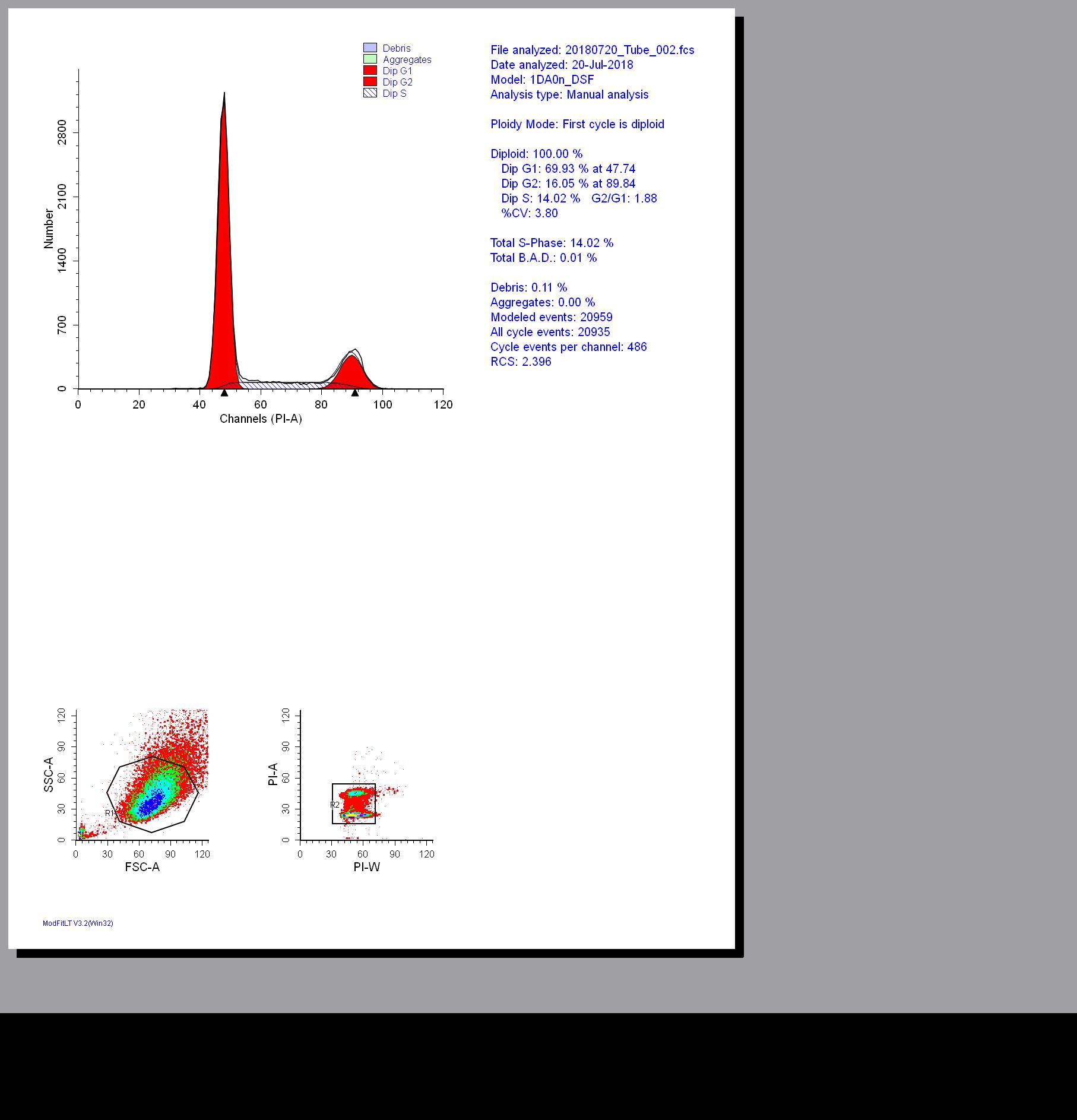

Supplement: Supplementary file 1 [file DataSheet_1.zip › Image 7.JPEG]

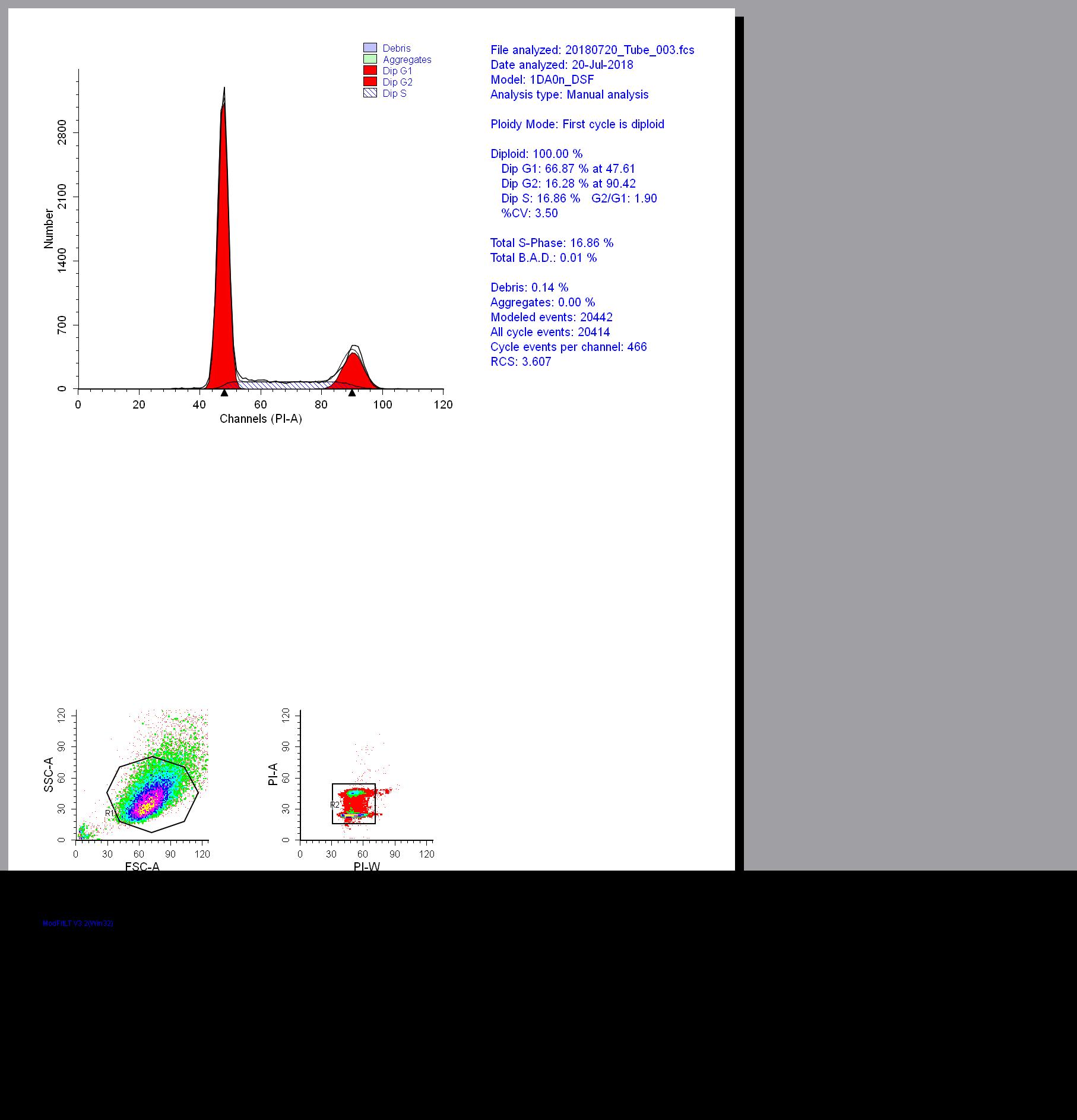

Supplement: Supplementary file 1 [file DataSheet_1.zip › Image 8.JPEG]

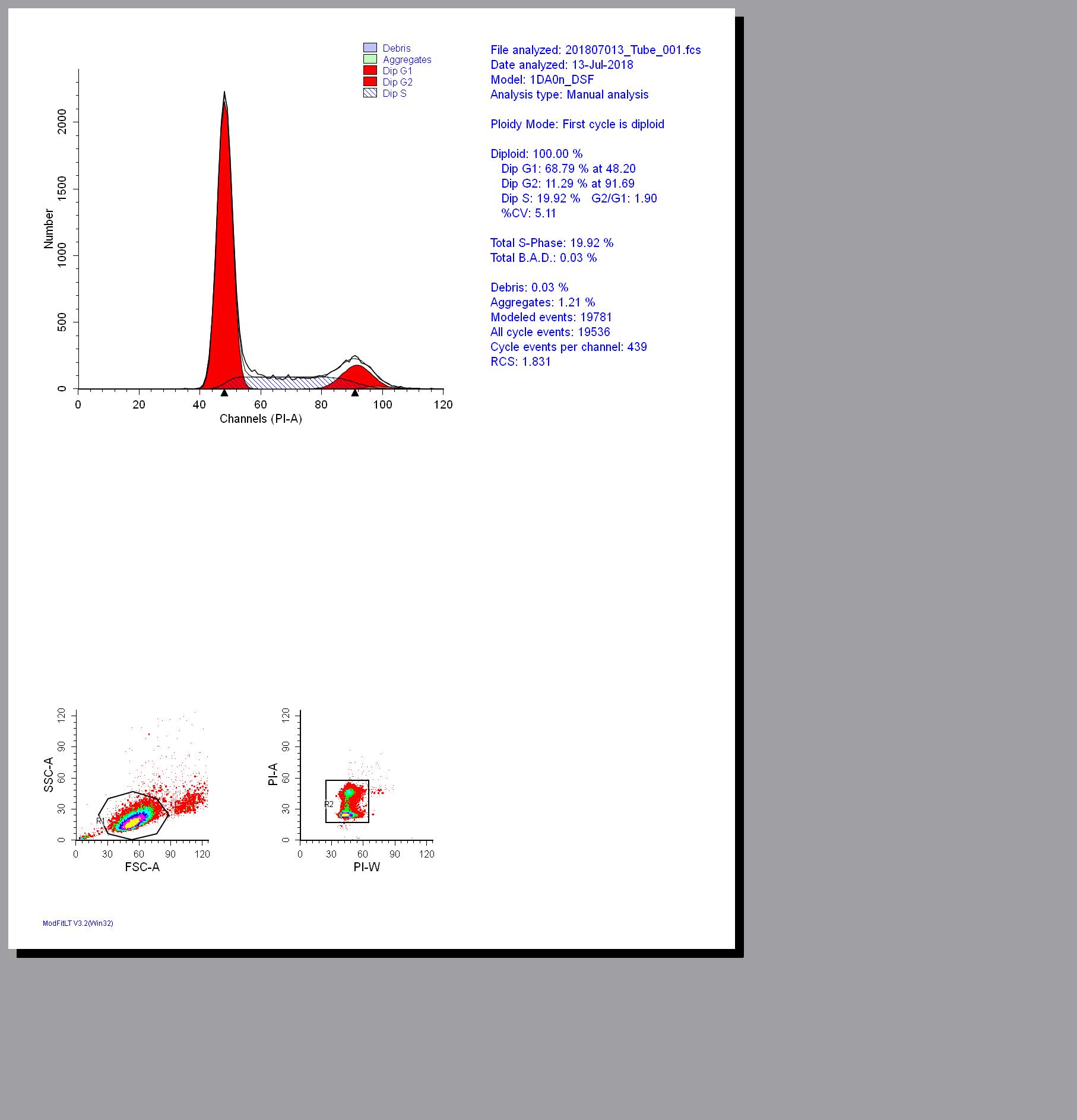

Supplement: Supplementary file 1 [file DataSheet_1.zip › Image 9.JPEG]
